# Supplementary material for: Oleanolic acid blocks the purine salvage pathway for cancer therapy by inactivating SOD1 and stimulating lysosomal proteolysis
Source: Mol Ther Oncolytics. 2021 Aug 28;23:107–23. doi: 10.1016/j.omto.2021.08.013 (PMC8505360; doi:10.1016/j.omto.2021.08.013)
Supplement: Document 1. Supplemental materials and methods, Tables S1–S5, and Figures S1–S5 [file mmc1.pdf]

OMTO, Volume *23*

Supplemental information

Oleanolic acid blocks the purine salvage pathway for cancer therapy by inactivating SOD1 and stimulating lysosomal proteolysis

Dan Liu, Xing Jin, Guanzhen Yu, Mingsong Wang, Lei Liu, Wenjuan Zhang, Jia Wu, Fengying Wang, Jing Yang, Qin Luo, Lili Cai, Xi Yang, Xisong Ke, Yi Qu, Zhenye Xu, Lijun Jia, and Wen-Lian Chen

Supplemental information

Supplemental Materials and Methods

Apoptosis assay

A549 and MDA-MB-231 cells were seeded into 6-well plates at a density of 1.5×10^5/well, and then treated by 200 μM OA for 0, 24, or 48 hours. Spent medium of each well was collected individually to harvest cells by centrifuging at 1500 rpm for 5 minutes, while adherent cells of each well were digested with 0.5% trypsin and harvested. For each well, cells from spent medium and adherent cells were combined together and then washed by PBS for 2 times. Finally, cell apoptosis assay was implemented on a flow cytometer (BD FACSVerse™, New Jersey, USA) using an AnnexinV-FITC and PI Apoptosis Kit (Yuheng Biotechnology, Suzhou, China) according to the manufacturer’s instructions.

Cell cycle analysis

A549 and MDA-MB-231 cells were seeded into 6-well plates at a density of 1.5×10^5/well and treated by 200 μM OA for 0, 24, or 48 hours. After removal of the spent medium, OA-treated cells and control cells were harvested and fixed with 70% ethanol at -20 °C overnight. Subsequently, the fixed cells were stained with propidium iodide (PI) (Sigma, St. Louis, MO, USA) at a concentration of 36 mg/mL at 37 °C for 15 minutes. Next, these cells were used for fluorescence activated cell sorting (FACS) analysis by a flow cytometer (BD FACSVerse™, New Jersey, USA). Data were analyzed with FlowJo 7.6 software.

Co-immunoprecipitation and ubiquitination assays of HGPRT

For the co-IP assay of HGPRT, A549 cells treated by vehicle or 200 μM OA for 8 hours were harvested and lysed in NP40 buffer (Beyotime, Shanghai, China) for 30 minutes on ice, and then centrifuged at 13,000 ×g for 15 minutes at 4 ℃. The lysates were incubated with anti-rabbit IgG (Cell Signaling Technology, Boston, Massachusetts, USA) or HGPRT antibody (ab109021, Abcam, Cambridge, UK) at 4 ℃ with rocking overnight. Subsequently, the lysates were incubated with protein A/G PLUS-Agarose beads (Santa Cruz, Dallas, Texas, USA) at 4 ℃ for 2 hours. The proteins binding to the beads were washed for three times by using NP40 buffer and then boiled for 10 minutes at 95 ℃, followed by SDS-PAGE, and finally analyzed by immunoblotting with primary antibodies against human hypoxanthine-guanine phosphoribosyltransferase (ab109021, Abcam, Cambridge, UK) and ubiquitin (Santa Cruz, Dallas, Texas, USA).

Table S1. Clinical characteristics of lung cancer patient cohort 1.

	Lung adenocarcinoma (n=5)
Age, years	
Median	64
Range	58-68
Gender, no. (%)	
Male	2（40）
Female	3（60）
TNM stage, no. (%)	
1	2（40）
2	2（40）
3	1（20）

Table S2. Clinical characteristics of lung cancer patient cohort 2.

	Lung adenocarcinoma (n=17)	Lung squamous cell carcinoma (n=17)
Age, years		
Median	60	60
Range	41-75	43-78
Gender, no. (%)		
Male	11（64.7）	14（82.3）
Female	6（35.3）	3（17.6）
TNM stage, no. (%)		
1	5（29.4）	
2	6（35.3）	7（41.2）
3	4（23.5）	5（29.4）
4	1（5.9）	1（5.9）
Unclassified	1（5.9）	4（23.5）

Table S3. Clinical characteristics of breast cancer patient cohort.

	Luminal-like (n=10)	Triple Negative (n=1)	HER2 positive (n=1)	Unclassified (n=8)
Age, years				
Median	52	49	59	47
Range	36-72	49	59	37-69
Gender, no. (%)				
Female	10（100）	1（100）	1（100）	8（100）
TNM stage, no. (%)				
1	3（30）	0（0）	0（0）	0（0）
2	5（50）	1（100）	1（100）	3（37.5）
3	2（20）	0（0）	0（0）	4（50.0）
4	0（0）	0（0）	0（0）	0（0）
Unclassified	0（0）	0（0）	0（0）	1（12.5）

Table S4. Clinical characteristics of lung cancer patient cohort 3.

	Lung adenocarcinoma (n=21)	Lung squamous cell carcinoma (n=13)
Age, years		
Median	60	63
Range	42-79	58-77
Gender, no. (%)		
Male	10（47.6）	11（84.6）
Female	11（52.4）	2（15.4）
TNM stage, no. (%)		
1	1（4.8）	0
2	13（61.9）	10（76.9）
3	6（28.5）	3（23.1）
4	1（4.8）	0

Table S5. Sequences of gene-specific q-PCR primers in this study.

Gene	NCBI Lous ID	Forward primer sequence (5'-3')	Reverse primer sequence (5'-3')
HPRT1	NM_3251	CCTGGCGTCGTGATTAGTGAT	AGACGTTCAGTCCTGTCCATAA
NT5E	NM_4907	AAGGACTGATCGAGCCACTC	GGAAGTGTATCCAACGATTCCCA
ACTIN	NM_001101.3	CACTCTTCCAGCCTTCCTTC	GTACAGGTCTTTGCGGATGT

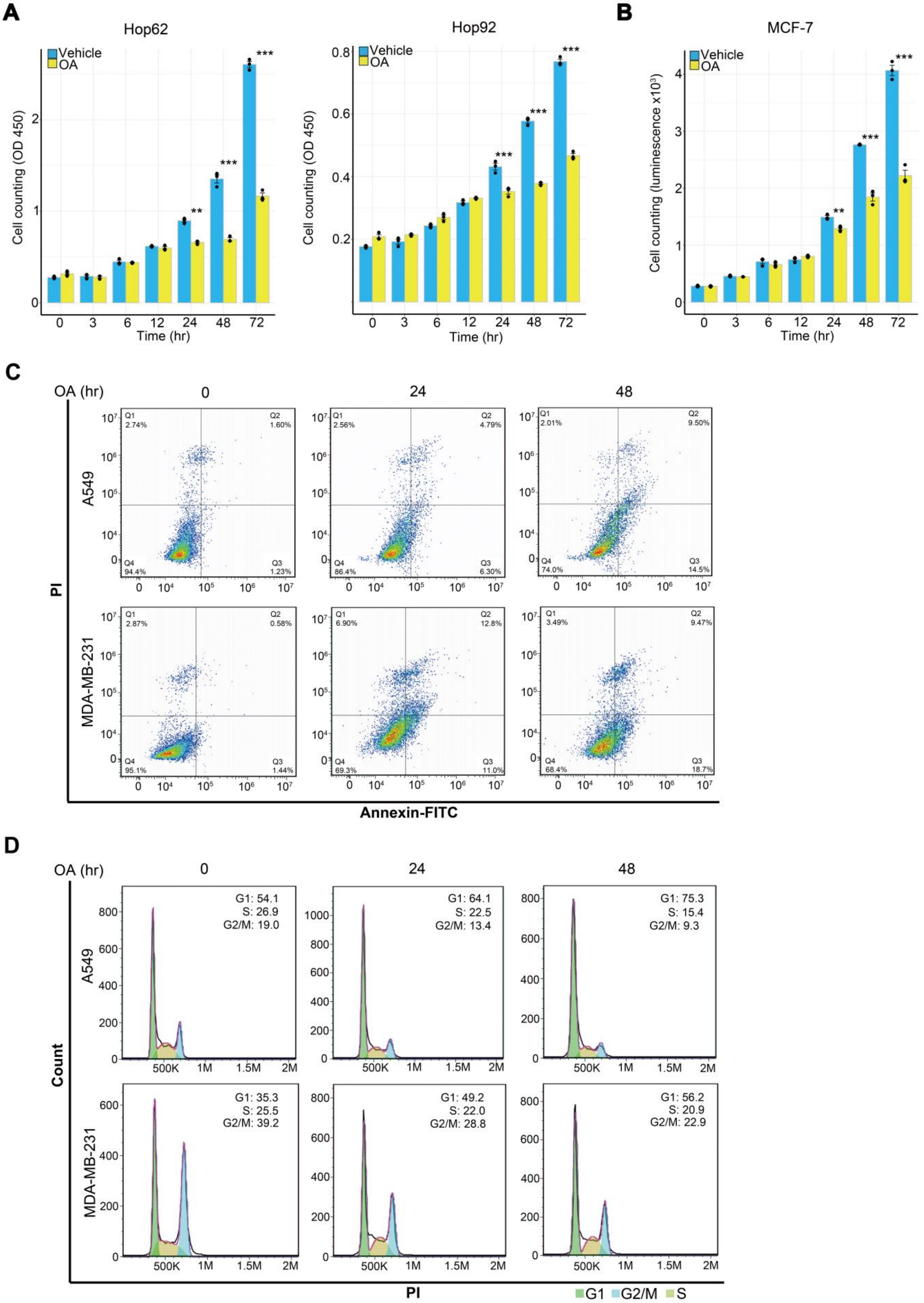
A
Hop62
Vehicle
OA
Cell counting (OD 450)
Time (hr)
Hop92
B
MCF-7
Cell counting (luminescence x10³)
C
OA (hr)
A549
MDA-MB-231
PI
Annexin-FITC
Q1 2.74%
Q2 1.60%
Q4 94.4%
Q3 1.23%
Q1 2.56%
Q2 4.79%
Q4 86.4%
Q3 6.30%
Q1 2.01%
Q2 9.50%
Q4 74.0%
Q3 14.5%
Q1 2.87%
Q2 0.58%
Q4 95.1%
Q3 1.44%
Q1 6.90%
Q2 12.8%
Q4 69.3%
Q3 11.0%
Q1 3.49%
Q2 9.47%
Q4 68.4%
Q3 18.7%
D
G1: 54.1 S: 26.9 G2/M: 19.0
G1: 64.1 S: 22.5 G2/M: 13.4
G1: 75.3 S: 15.4 G2/M: 9.3
G1: 35.3 S: 25.5 G2/M: 39.2
G1: 49.2 S: 22.0 G2/M: 28.8
G1: 56.2 S: 20.9 G2/M: 22.9
Count
PI
G1 G2/M S

Figure S1 Oleanolic acid impedes cancer cell growth and induces cell apoptosis and cell cycle arrest *in vitro*.

(A-B) The time-course influence of 200 μM oleanolic acid (OA) on the cell proliferation of two lung cancer cell lines Hop92 and Hop62, and one breast cancer cell line MCF-7. **(C)** The time course impact of 200 μM OA on cell apoptosis of A549 and MDA-MB-231 cells. **(D)** The time course effect of 200 μM OA on cell cycle of A549 and MDA-MB-231 cells.

Error bars represent mean ± SEM. $^{*}p < 0.05$, $^{**}p < 0.01$, $^{***}p < 0.001$, Student's t test.

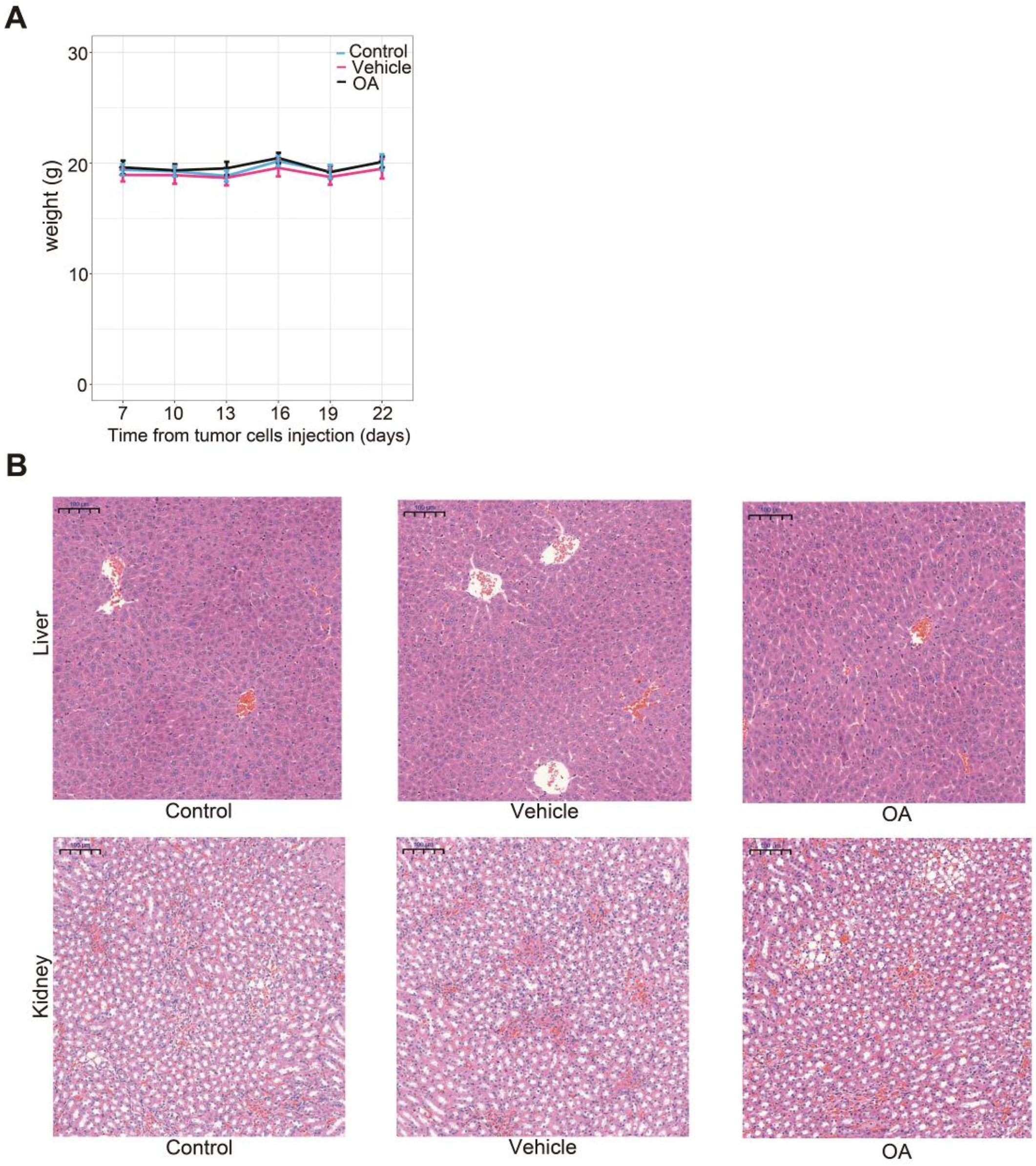

Figure S2. The influence of oleanolic acid treatment on mouse body weight and histological structure of liver and kidney.

(A) Body weight of healthy control mice, A549 tumor-bearing mice treated with vehicle, and A549 tumor-bearing mice treated with oleanolic acid (OA, 120 mg/kg/day). Error bars represent mean ± SEM. **(B)** Histological structure of liver and kidney tissues from healthy control mice, A549 tumor-bearing mice treated with vehicle, and A549 tumor-bearing mice treated with OA (120 mg/kg/day). Scale bars, 100 µm.

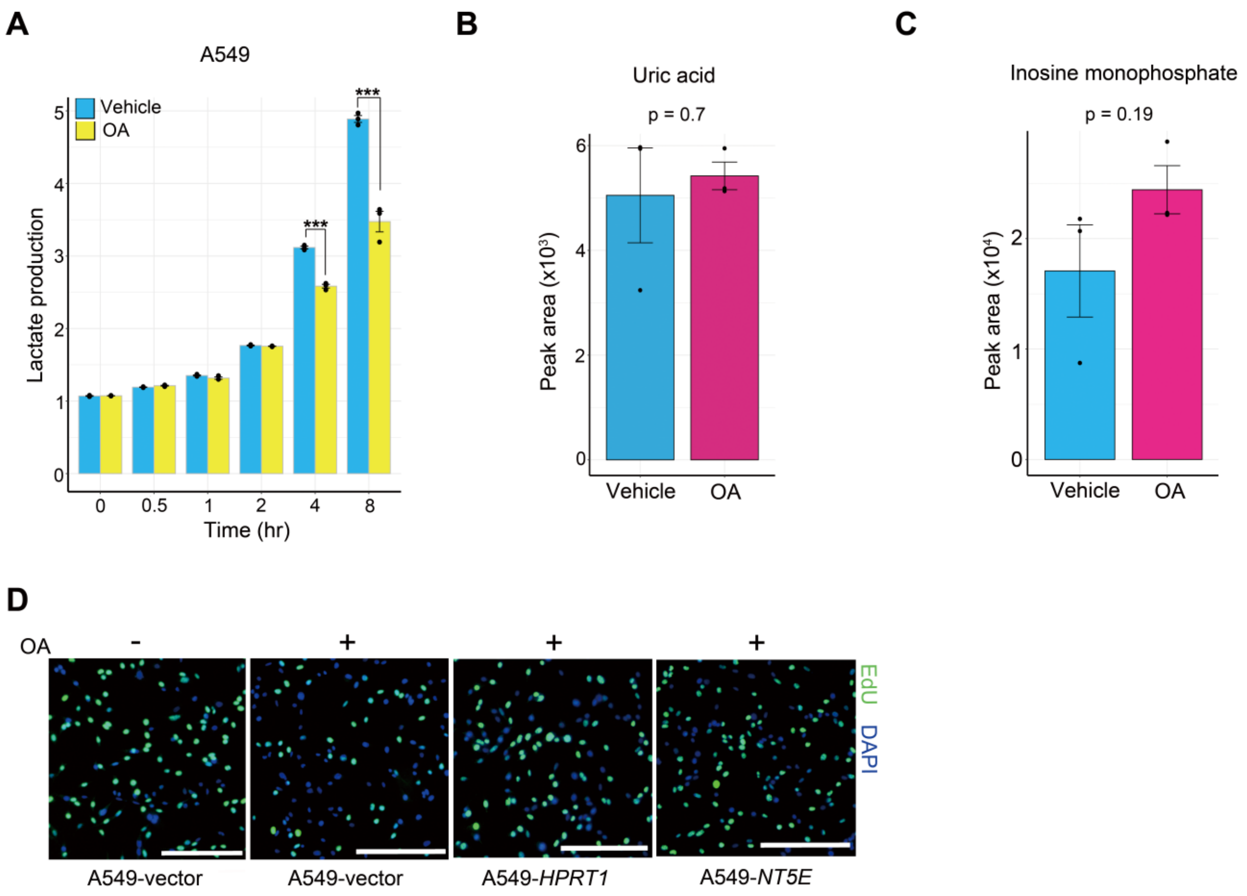

Figure S3. The impact of oleanolic acid treatment on cancer cell metabolism and the rescue effect of enforced expression of *HPRT1* or *NT5E* on DNA synthesis suppressed by oleanolic acid in A549 cells.

(A) The time course assay of lactate production of A549 cells treated by 200 μM oleanolic acid (OA). **(B)** Uric acid generation between A549 cells treated by vehicle (1/1000 DMSO) and A549 cells treated by 200 μM OA for 8 hours. **(C)** Inosine monophosphate (IMP) production between A549 cells treated by vehicle (1/1000 DMSO) and A549 cells treated by 200 μM OA for 8 hours. **(D)** DNA synthesis was remarkably refrained in A549-vector cells treated by 200 μM OA for 8 hours as compared to A549-vector cells treated by vehicle. Of note, impaired DNA synthesis elicited by OA was overtly rescued by forced expression of *HPRT1* or *NT5E*. DNA synthesis was measured by EdU incorporation (green), while cell nuclei were stained by DAPI (blue). Scale bars, 100 μm.

Error bars represent mean ± SEM. $^{*}p < 0.05$, $^{**}p < 0.01$, $^{***}p < 0.001$, Student's t test.

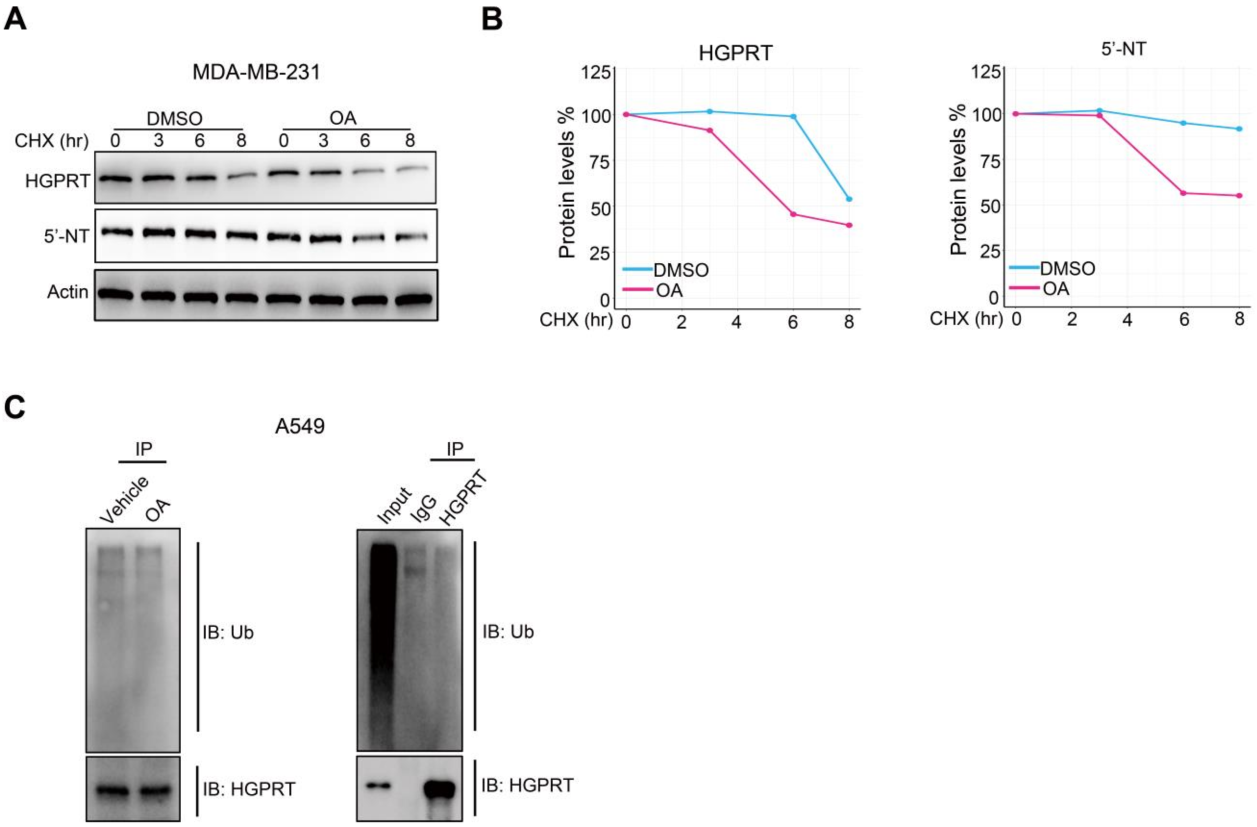

Figure S4. Oleanolic acid expedites the degradation of PSP enzymes via lysosomal pathway but not ubiquitination pathway.

(A-B) The time-course influence of 200 μM oleanolic acid (OA) treatment on HGRPT and 5'-NT degradation when using cycloheximide (CHX, 50 μg/mL) to block protein synthesis in MDA-MB-231 cells. The curves on the right side (B) displaying the quantification results of the protein levels across different time points. **(C)** The effect of 200 μM OA treatment for 8 hours on the ubiquitination status of HGPRT in A549 cells. The affinity and specificity of the anti-HGPRT antibody to cellular HGPRT protein was validated as showed by the immunoblots on the right side.

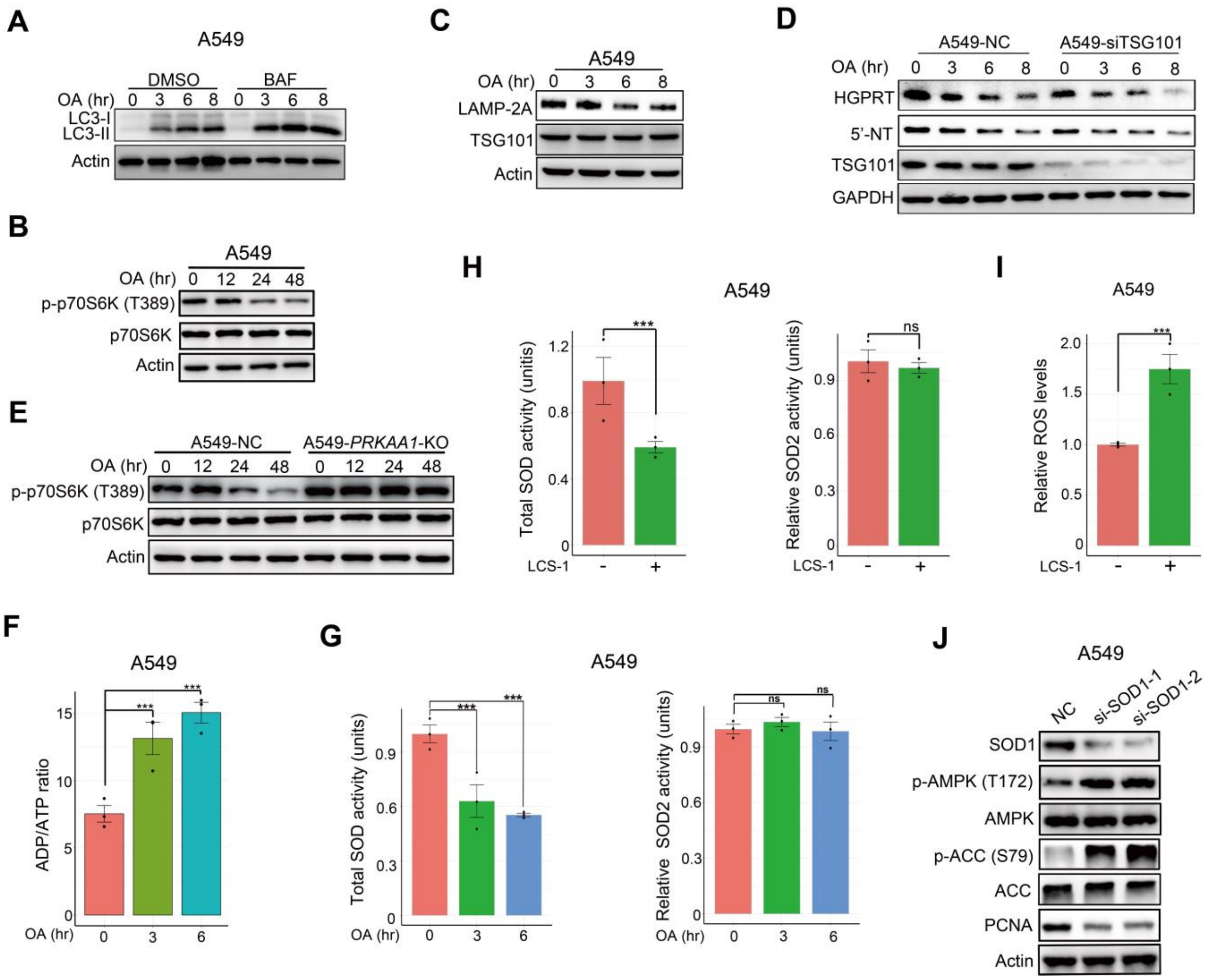

Figure S5. Stimulation of macroautophagy but not microautophagy and chaperone-mediated autophagy by oleanolic acid treatment, and the underlying mechanism of oleanolic acid-activated macroautophagy.

(A) Western blot showing the time course of a lysosome inhibitor bafilomycin A1 (BAF, 0.08 μM) treatment effect on LC3-I/II levels in A549 cells under the condition of 200 μM OA treatment. **(B)** Western blot showing the time course of the impact of 200 μM OA treatment on the expression of phospho-p70S6K (T389) and p70S6K in A549 cells. **(C)** Western blot showing the time course of the effect of 200 μM OA treatment on the expression of TSG101 and LAMP-2A in A549 cells. TSG101 is a microautophagy-related protein, while LAMP-2A is a chaperone-mediated

autophagy-related protein. **(D)** Western blot showing the time course of the effect of 200 μM OA treatment on the expression of HGPRT and 5'-NT between control A549 cells and A549 cells with TSG101 silencing. TSG101 knockdown was used to inhibit microautophagy. **(E)** Western blot showing the time course of the effect of 200 μM OA treatment on the expression of phosho-p70S6K (T389) and p70S6K in A549 cells with and without *PRKAA1*-KO. **(F)** The time course of the effect of 200 μM OA treatment on ADP/ATP ratio in A549 cells. **(G)** The time course of the influence of 200 μM OA treatment on the activities of total superoxide dismutases (SODs) and SOD2 in A549 cells. **(H)** The impact of a SOD1 inhibitor LCS-1 (5 μM**)** treatment for 6 hours on the activity of total SODs and SOD2 in A549 cells. **(I)** Measurement of ROS generation in A549 cells with or without LCS-1 (5 μM) treatment for 6 hours. **(J)** Western blot showing the impact of SOD1 silencing on the expression of SOD1, phospho-AMPK (T172), AMPK, phospho-ACC (S79), ACC and PCNA in A549 cells. The cells were treated by siRNAs for 72 hours. NC, nontarget control siRNA sequence. Error bars represent mean ± SEM. *p < 0.05, **p < 0.01, ***p < 0.001, Student's t test. Ns, no significance.
