# Supplementary material for: Oleanolic acid blocks the purine salvage pathway for cancer therapy by inactivating SOD1 and stimulating lysosomal proteolysis
Source: Mol Ther Oncolytics. 2021 Aug 28;23:107–23. doi: 10.1016/j.omto.2021.08.013 (PMC8505360; doi:10.1016/j.omto.2021.08.013)
Supplement: Document 2. Article plus supplemental information [file mmc2.pdf]

Molecular Therapy
Oncolytics
Original Article

Oleanolic acid blocks the purine salvage pathway for cancer therapy by inactivating SOD1 and stimulating lysosomal proteolysis

Dan Liu,[1,7] Xing Jin,[1,7] Guanzhen Yu,[1] Mingsong Wang,[2] Lei Liu,[3] Wenjuan Zhang,[4] Jia Wu,[1] Fengying Wang,[1] Jing Yang,[1] Qin Luo,[1] Lili Cai,[1] Xi Yang,[1] Xisong Ke,[5] Yi Qu,[5] Zhenye Xu,[6] Lijun Jia,[1] and Wen-Lian Chen[1]

[1]Cancer Institute, Longhua Hospital, Shanghai University of Traditional Chinese Medicine, Shanghai 200032, China; [2]Department of Thoracic Cardiovascular Surgery, Xinhua Hospital of Shanghai Jiaotong University School of Medicine, Shanghai 200092, China; [3]Department of Thoracic Surgery, the Affiliated Tumor Hospital of Nantong University, Nantong 226361, China; [4]Cancer Institute, Fudan University Shanghai Cancer Center, Shanghai 200032, China; [5]Center for Chemical Biology, Shanghai University of Traditional Chinese Medicine, Shanghai 201203, China; [6]Department of Oncology, Longhua Hospital, Shanghai University of Traditional Chinese Medicine, Shanghai 200032, China

Metabolic reprogramming is a core hallmark of cancer and is key for tumorigenesis and tumor progression. Investigation of metabolic perturbation by anti-cancer compounds would allow a thorough understanding of the underlying mechanisms of these agents and identification of new anti-cancer targets. Here, we demonstrated that the administration of oleanolic acid (OA) rapidly altered cancer metabolism, particularly suppressing the purine salvage pathway (PSP). PSP restoration significantly opposed OA-induced DNA replication and cell proliferation arrest, underscoring the importance of this pathway for the anti-cancer activity of OA. Hypoxanthine-guanine phosphoribosyltransferase (HGPRT) and 5′-nucleotidase (5′-NT), two metabolic enzymes essential for PSP activity, were promptly degraded by OA via the lysosome pathway. Mechanistically, OA selectively targeted superoxide dismutase 1 (SOD1) and yielded reactive oxygen species (ROS) to activate the AMP-activated protein kinase (AMPK)/mammalian target of rapamycin complex 1 (mTORC1)/macroautophagy pathway, thus eliciting lysosomal degradation of HGPRT and 5′-NT. Furthermore, we found that the PSP was overactivated in human lung and breast cancers, with a negative correlation with patient survival. The results of this study elucidated a new anti-cancer mechanism of OA by restraining the PSP via the SOD1/ROS/AMPK/mTORC1/macroautophagy/lysosomal pathway. We also identified the PSP as a new target for cancer treatment and highlighted OA as a potential therapeutic agent for cancers with high PSP activity.

INTRODUCTION

Cancer is a devastating disease and the second-leading cause of deaths worldwide.[1] Breakthroughs in fundamental and clinical research have allowed the development of cancer treatment approaches including surgery, chemotherapy, radiotherapy, targeted therapy, and immunotherapy.[2] However, the therapeutic outcomes and prognoses of patients with cancer remain unsatisfactory.[2] Therefore, new therapeutic agents and targets must be identified. Natural compounds have long been recognized as valuable sources for anti-cancer drug development.[3,4] For instance, bioactive ingredients in triterpenoid species with anti-cancer activity have been discovered.[5] Oleanolic acid (OA) is one such bioactive ingredient that has been well studied. This compound occurs in various natural plants that are extensively used in anti-cancer Chinese herbal medicine formulas, such as *Ligustrum lucidum* Ait. and *Epimedii folium*.[6,7] OA tablets have been approved in China for adjuvant therapy in patients with acute or chronic hepatitis.[8] Recently, OA has been reported to show anti-cancer activity by modulating various oncogenic signaling pathways, including the AMP-activated protein kinase (AMPK)/mammalian target of rapamycin complex 1 (mTORC1), ERK/Nrf2/reactive oxygen species (ROS), and phosphatidylinositol 3-kinase (PI3K)/AKT/mammalian target of rapamycin (mTOR) signaling pathways.[9,10] However, further investigation is needed to comprehensively understand the anti-cancer mechanisms of this natural compound.

Recent studies have demonstrated that metabolic reprogramming is a core hallmark of cancer and is involved in tumorigenesis and tumor progression.[11] In response to the excessive requirement for building blocks and energy, cancer cells rewire their glycolysis and truncate the tricarboxylic acid (TCA) cycle by expediting glucose and glutamine utilization.[12,13] Additionally, alternative metabolic fuels, including fructose, lactate, and branched-chain amino acids, are also utilized by cancer cells to sustain the metabolic activities of

Received 22 February 2021; accepted 24 August 2021;
https://doi.org/10.1016/j.omto.2021.08.013.

[7]These authors contributed equally

Correspondence: Wen-Lian Chen, PhD, Cancer Institute, Longhua Hospital, Shanghai University of Traditional Chinese Medicine, Building 12 RM 607, No. 725 South Wanping Rd., Shanghai 200032, China.
E-mail: chenwl8412@shutcm.edu.cn
Correspondence: Professor Lijun Jia, PhD, Cancer institute, Longhua Hospital, Shanghai University of Traditional Chinese Medicine, Building 12 RM 601, No. 725 South Wanping Rd., Shanghai 200032, China.
E-mail: ljjia@shutcm.edu.cn

This is an open access article under the CC BY license (http://creativecommons.org/licenses/by/4.0/).

glycolysis, TCA cycle, and synthesis of nonessential amino acids, respectively.[14–16] Nucleotide synthesis is also hyperactive in cancer cells to produce abundant genetic material, including DNA and RNA.[17,18] OA reportedly restrained glycolysis and lipid biosynthesis in cancer cells.[9] However, the effects of OA on the metabolism of cancer cells and its major downstream metabolic targets remain to be elucidated.

Mass spectrometry-based metabolomic profiling is sensitive and robust for the simultaneous identification of some small-molecule metabolites from various samples.[19] This method has been extensively applied in cancer research to ascertain the essential metabolic pathways that promote tumor progression.[14,20] The present study used a gas chromatography-time-of-flight mass spectrometry (GC-TOFMS)-based approach to detect the effects of OA on the metabolism of cancer cells and to identify the key metabolic pathways targeted by this agent. We then performed experiments to explore the underlying mechanism of OA in modulating these identified metabolic targets in cancer cells.

RESULTS

OA restrains cancer cell growth *in vitro* and *in vivo*

Previous studies have reported that OA inhibits the rapid growth of various cancer cell lines.[9,10,21] However, the time point at which OA begins to show this inhibitory effect remains unclear. Three lung cancer cell lines (A549, Hop62, and Hop92) and two breast cancer cell lines (MDA-MB-231 and MCF-7) were selected for investigation. First, cell viability and colony formation assays were performed to verify the inhibitory effects of OA. OA repressed cancer cell proliferation and colony growth in a dose-dependent manner (Figures 1A, 1B, and 1C; Figures S1A and S1B). Subsequently, we conducted a time course experiment using an OA concentration of 200 μM as reported previously.[9] As shown in Figure 1D, OA significantly suppressed cell growth beginning at 48 and 24 h after treatment initiation in A549 and MDA-MB-231 cells, respectively. The expression of the well-known cell proliferation marker proliferating cell nuclear antigen (PCNA) consistently started to decrease until 48 h of treatment in A549 cells and 24 h of treatment in MDA-MB-231 cells, with a stronger decrease observed in late-stage OA treatment (Figure 1E). Collectively, these *in vitro* data demonstrated the inhibitory effect of OA on cancer cell growth over a long treatment period.

To elucidate the underlying mechanism by which OA curtailed cancer cell growth, we performed cell cycle and apoptosis assays. The results showed that 200 μM OA treatment elicited a time course of cell apoptosis and G1 phase arrest in A549 and MDA-MB-231 cells (Figures S1C and S1D), indicating that OA impaired cancer cell growth by inducing cell apoptosis and hindering cell cycle progression.

Next, we explored whether OA could also impede tumor growth *in vivo*. OA was orally administered to mice harboring tumor xenografts. The results showed that OA administration significantly attenuated tumor xenograft growth in A549 and MDA-MB-231 cells (Figures 1F-1I). Of note, mouse body weight and the histological structures of the liver and kidney were not obviously altered by OA treatment, indicating the negligible side effects of OA for *in vivo* use (Figure S2).

OA rapidly suppresses PSP *in vitro* and *in vivo*

Metabolic reprogramming plays an essential role in tumorigenesis and tumor progression.[11,22,23] We hypothesized that OA exerts its anti-cancer effects by modulating key metabolic pathways in cancer cells. To this end, we conducted a metabolomic study to elucidate the major metabolic pathways targeted by OA. An essential issue was to determine the time point of OA treatment for metabolomic surveys. As mentioned above, OA treatment for <48 h did not influence A549 cell growth (Figures 1D and 1E). In addition, our preliminary study showed that the production of lactate, a targeted metabolite of OA,[9] was downregulated by OA treatment for 6 h, whereas a stronger reduction of this metabolite was observed after OA treatment for 8 h (Figure S3A). Thus, we harvested A549 cells treated with OA for 8 h and control A549 cells treated with vehicle for the same time for metabolomic profiling. A principal component analysis model fitted with metabolomics data revealed altered metabolism in the OA-treated A549 cells (Figure 2A). At the metabolite level, a total of 163 metabolites were identified, 18 of which were significantly altered by OA treatment (Bonferroni-adjusted $p < 0.05$) (Figure 2B). Subsequently, we included all detected metabolites to calculate the differential abundance scores of each metabolic pathway with an algorithm described previously.[24] The results showed that the purine salvage pathway (PSP) was the most severely inhibited pathway after OA treatment for 8 h (Figure 2C). To ascertain whether the reduction of PSP metabolites induced by OA treatment was caused by the expedited catabolism of these compounds, we compared the levels of uric acid, the end product of purine ring degradation, between control and OA-treated A549 cells. The results showed that OA treatment did not dramatically perturb the abundance of uric acid (Figure S3B), indicating that OA did not accelerate the catabolism of PSP metabolites. Furthermore, we assessed whether OA treatment repressed *de novo* purine synthesis, which could cause compensatory upregulation of the PSP to increase the conversion of nucleosides to nucleotides, thereby downregulating PSP metabolites in cancer cells. The abundance of inosine monophosphate (IMP), the first purine nucleotide product of *de novo* purine synthesis, did not significantly change in A549 cells after OA treatment ($p = 0.19$) (Figure S3C). Thus, OA did not modify the *de novo* purine synthesis of cancer cells.

As illustrated by the schematic of the PSP, OA treatment significantly downregulated inosine, hypoxanthine, and adenosine, whereas the homeostasis of these purine metabolites was regulated by two key metabolic enzymes, hypoxanthine-guanine phosphoribosyltransferase (HGPRT) encoded by *HPRT1* and 5′-nucleotidase (5′-NT) encoded by *NT5E* (Figure 2D). To verify whether OA treatment inhibited PSP activity, we measured the changes in HGPRT and 5′-NT activity. We found that 200 μM OA treatment for 8 h markedly reduced the activity of both enzymes in A549 cells (Figure 2E).

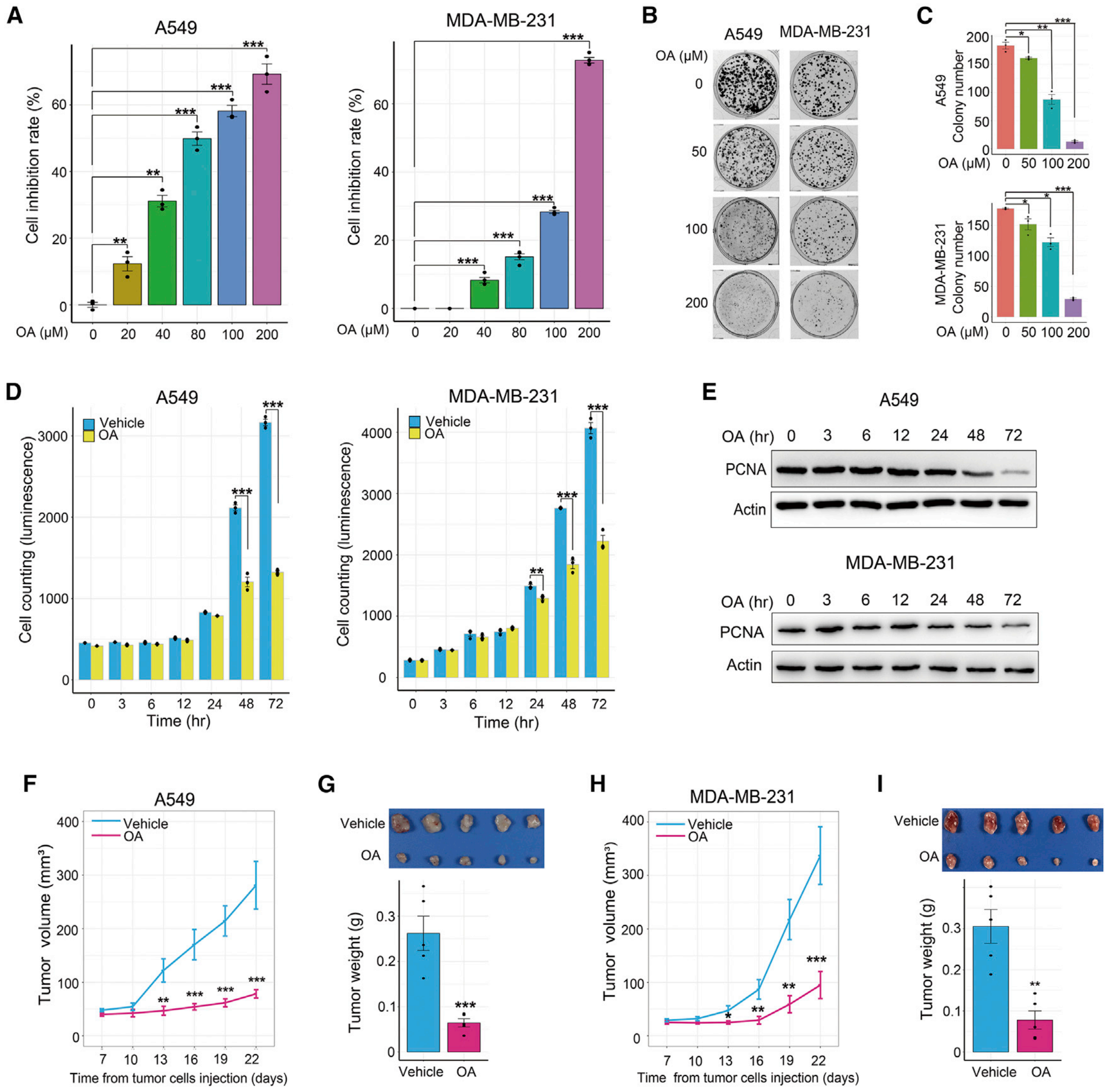

Figure 1. OA hinders cancer cell growth *in vitro* and *in vivo*

(A and B) The influence of different OA concentrations on cell proliferation (A) and colony formation (B) in A549 and MDA-MB-231 cells. In the cell proliferation assay, the cells were treated for 72 h and viable cells were counted with an ATPlite kit. The cell inhibition rate (%) was defined as (mean luminescence value of control cells − luminescence value of cells treated by different OA concentrations)/mean luminescence value of control cells × 100. In the cell colony formation assays, A549 and MDA-MB-231 cells were treated for 14 and 18 days, respectively. (C) Quantitative measurements of colony formation in A549 and MDA-MB-231 cells treated with different OA concentrations. (D) Time course assay of cell proliferation in A549 and MDA-MB-231 cells treated with 200 μM OA or vehicle (DMSO diluted at a ratio of 1:1,000). Viable cells were counted with an ATPlite kit. (E) Time course assay of proliferating cell nuclear antigen (PCNA) expression in A549 and MDA-MB-231 cells treated with 200 μM OA or vehicle (DMSO diluted at a ratio of 1:1,000) for 72 h. (F–I) Impact of oral OA (120 mg/kg/day) administration on subcutaneous tumor xenograft growth of A549 (F and G) and MDA-MB-231 (H and I) cells. Error bars represent mean ± SEM. *p < 0.05, **p < 0.01, ***p < 0.001, Student's t test.

This result provided robust evidence to verify OA-induced restraint of PSP activity in cancer cells.

To confirm whether *in vivo* OA administration also inhibited the PSP, we carried out a metabolomic investigation of A549 tumor xenografts with or without the oral administration of OA. Consistent with *in vitro* findings, OA administration dramatically altered the metabolism of A549 xenografts (Figure 2F). Notably, the oral administration of OA dramatically restrained PSP activity, as demonstrated by the downregulated metabolites of this pathway, including hypoxanthine, inosine, and adenosine (Figure 2G). Thus, we concluded that the PSP is a key downstream target of OA both *in vitro* and *in vivo*.

PSP blockade is required for the anti-cancer activity of OA

We performed rescue assays to determine whether the PSP blockade by OA contributed to the anti-cancer activity of this herbal

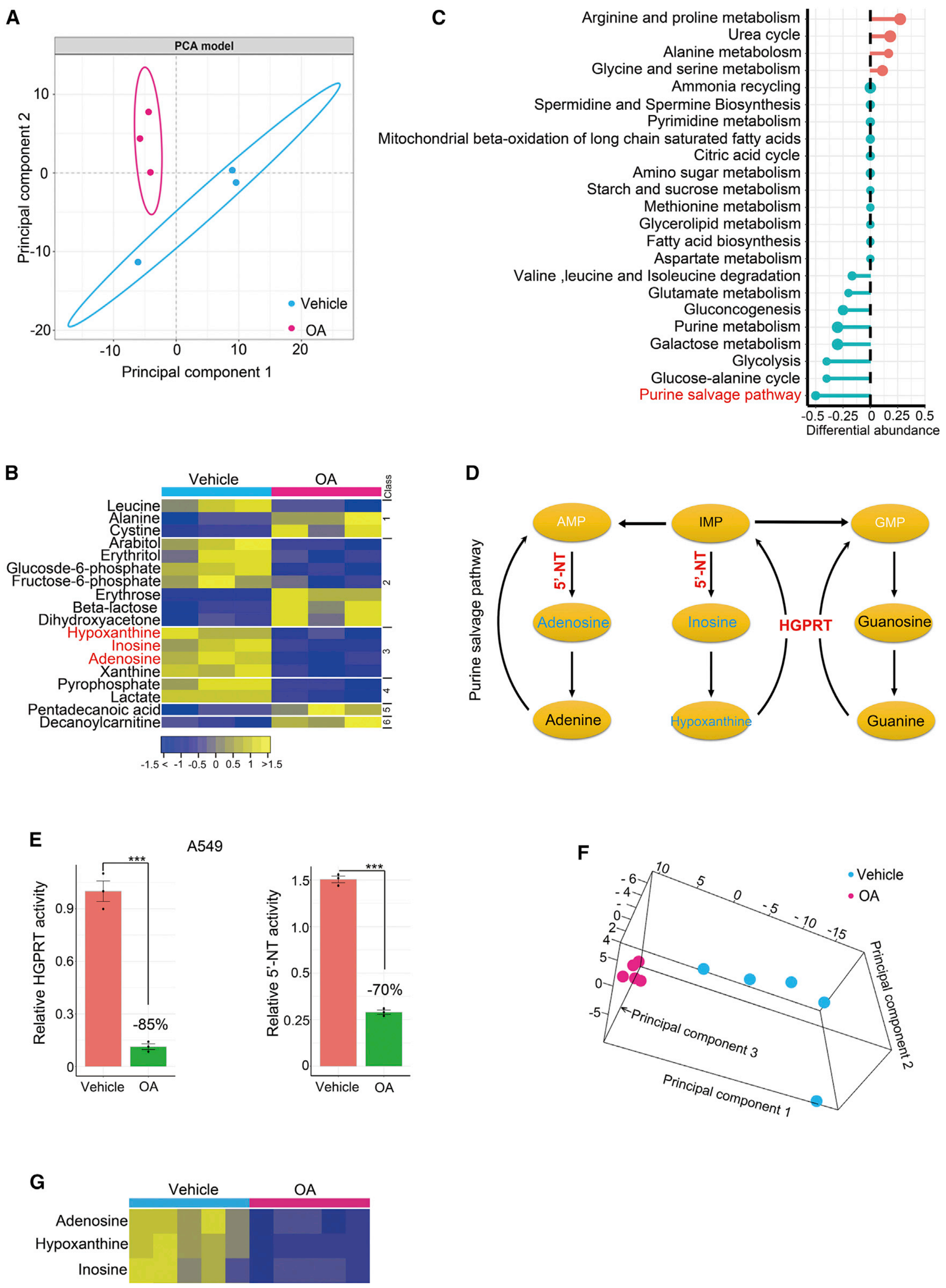

Figure 2. OA treatment quickly alters metabolism and restrains the PSP of cancer cells *in vitro* and *in vivo*

(A) Principal component analysis (PCA) score plot showing metabolic profiles of A549 cells treated with 200 μM OA and vehicle, respectively. The cells were treated for 8 h. (B) Heatmap showing differentially expressed metabolites between A549 cells treated with 200 μM OA or vehicle (DMSO diluted at a ratio of 1:1,000) for 8 h. The metabolites of

(legend continued on next page)

compound. Two parameters, DNA replication (measured by 5-ethynyl-2′-deoxyuridine [EdU] incorporation) and cell proliferation, were analyzed. OA treatment hindered EdU incorporation into A549 and MDA-MB-231 cells in a time-dependent manner (Figures 3A and 3B), indicating that OA repressed DNA replication in cancer cells. Notably, the exogenous addition of inosine or hypoxanthine restored EdU incorporation into OA-treated cells (Figures 3C–3F). Importantly, when the PSP was not disturbed by OA, exogenous supplementation with inosine or hypoxanthine did not alter EdU incorporation (Figures 3C–3F).

Subsequently, we noted that exogenous supplementation of inosine or hypoxanthine restored the growth of A549 and MDA-MB-231 cells suppressed by OA (Figures 3G and 3H). Correspondingly, both hypoxanthine and inosine restored PCNA expression in cancer cells treated with OA (Figures 3G and 3H). Notably, when the endogenous PSP was not disturbed by OA, the exogenous provision of hypoxanthine or inosine did not influence cancer cell proliferation or PCNA expression (Figures 3G and 3H). These findings indicated that PSP blockade was responsible for the anti-cancer activity of OA.

OA promptly downregulates two key metabolic enzymes of PSP via lysosomal proteolysis

As natural compounds can affect the activities of major protein degradation pathways and modulate the homeostasis of intracellular proteins,[25] we speculated that OA could rapidly downregulate HGPRT and 5′-NT, two key enzymes mentioned above, to repress the PSP. Indeed, the protein levels of HGPRT and 5′-NT in cancer cells were slightly decreased beginning at the 6th hour of OA treatment, with a much stronger reduction observed at the 8th hour (Figure 4A). Consistent with *in vitro* findings, the oral administration of OA also remarkably downregulated HGPRT and 5′-NT in A549 and MDA-MB-231 tumor xenografts (Figures 4B and 4C). To confirm the role of these two enzymes in controlling cancer cell proliferation, we conducted gene knockout (KO) assays and found that the simultaneous deletion of *HPRT1* and *NT5E* markedly reduced A549 cell proliferation (Figure 4D). Furthermore, overexpression of *HPRT1* or *NT5E* restored both DNA synthesis and cell propagation impaired by OA treatment (Figure 4E; Figure S3D).

Because of the importance of HGPRT and 5′-NT in the PSP activity of cancer cells, a key question was how OA regulates these two metabolic enzymes in cancer cells. First, we performed quantitative polymerase chain reaction assays to evaluate the effect of OA on the transcription of these two enzymes. The results showed that OA treatment for 8 h did not alter *HPRT1* and *NT5E* transcription (Figure 5A). Subsequently, we tested whether OA influenced the translation of these two enzymes from mRNA. Cycloheximide (CHX), a reagent that blocks the elongation phase of eukaryotic protein translation,[26] was used to treat cancer cells incubated with or without OA. We observed dramatically faster HGPRT and 5′-NT degradation by OA treatment when protein synthesis was blocked by CHX (Figure 5B; Figures S4A and S4B).

As the general protein degradation systems are proteasome-mediated and lysosome-mediated degradation,[27] we determined which pathway was activated by OA to accelerate HGPRT and 5′-NT degradation. We added the lysosome inhibitor chloroquine (CQ) and the proteasome inhibitor MG132[28] to A549 cells under OA treatment. We observed that CQ, but not MG132, blocked HGPRT and 5′-NT degradation induced by OA (Figure 5C), suggesting that OA activated lysosomal proteolysis to accelerate HGPRT and 5′-NT degradation. To verify this result, we used another lysosome inhibitor, bafilomycin A1 (BAF).[29] The results showed that the inhibition of lysosomal proteolysis by BAF also impeded OA-induced HGPRT and 5′-NT degradation (Figure 5D). Notably, OA treatment did not alter the ubiquitination of HGPRT in A549 cells (Figure S4C), indicating that OA did not trigger the degradation of the PSP enzyme via the ubiquitination pathway. Thus, our results demonstrated that OA reduces HGPRT and 5′-NT levels in cancer cells by activating lysosomal proteolysis.

OA selectively inactivates superoxide dismutase 1 to degrade HGPRT and 5′-NT via ROS/AMPK/mTORC1/macroautophagy pathway

Next, we determined which upstream pathway stimulated by OA delivered HGPRT and 5′-NT to the lysosomes for degradation. At least three pathways have been described to transport materials to lysosomes, including autophagy, endocytosis, and micropinocytosis.[30] Of note, the function of CQ and BAF mentioned above is to impair autophagosome-lysosome fusion, thus suppressing lysosome-mediated protein degradation.[28,29] In addition, AMPK is an important upstream modulator of autophagy[31] and is strongly activated by OA treatment.[9] Therefore, we assumed that OA could activate the AMPK/autophagy pathway to deliver HGPRT and 5′-NT to the lysosomes for degradation. Indeed, on treatment with OA in A549 cells, phosphorylated AMPK at Ser172, phosphorylated acetyl-CoA carboxylase 1 (ACC) at Ser79 (a substrate of AMPK), and the autophagosome forming marker LC3-II isoform increased over time (Figure 6A), indicating that OA activated both AMPK and autophagy. To

the PSP are highlighted in red. The metabolites were subclassified as follows: (1) amino acids, (2) carbohydrates, (3) nucleotides, (4) organic acids, (5) lipids including fatty acids, and (6) unclassified. (C) A pathway-based analysis of metabolic alterations between A549 cells treated with 200 μM OA or vehicle for 8 h. The differential abundance score revealed the average change for metabolites in a pathway. Scores of 0.5 and −0.5 indicated increases or decreases in all detected metabolites in a pathway, respectively. (D) Scheme representing the metabolites and metabolic enzymes in the PSP. Metabolites significantly downregulated by OA treatment for 8 h are highlighted in deep sky blue, and metabolites with no significant alteration by OA treatment are highlighted in black. Metabolites participating in the pathway but not detected in the study are highlighted in white. Two enzymes, HGPRT and 5′-NT, responsible for the generation of metabolites perturbed by OA, are underlined in red. (E) Enzyme activities of HGPRT and 5′-NT in A549 cells treated with 200 μM OA or vehicle (DMSO diluted at a ratio of 1:1,000) for 8 h. (F) PCA score plot showing the impact of oral OA administration on metabolism of A549 tumor xenografts. (G) Three metabolites differentially expressed between A549 tumor xenografts with oral OA (n = 5) or vehicle (n = 5) administration. Error bars represent mean ± SEM. *$p < 0.05$, **$p < 0.01$, ***$p < 0.001$, Student's t test.

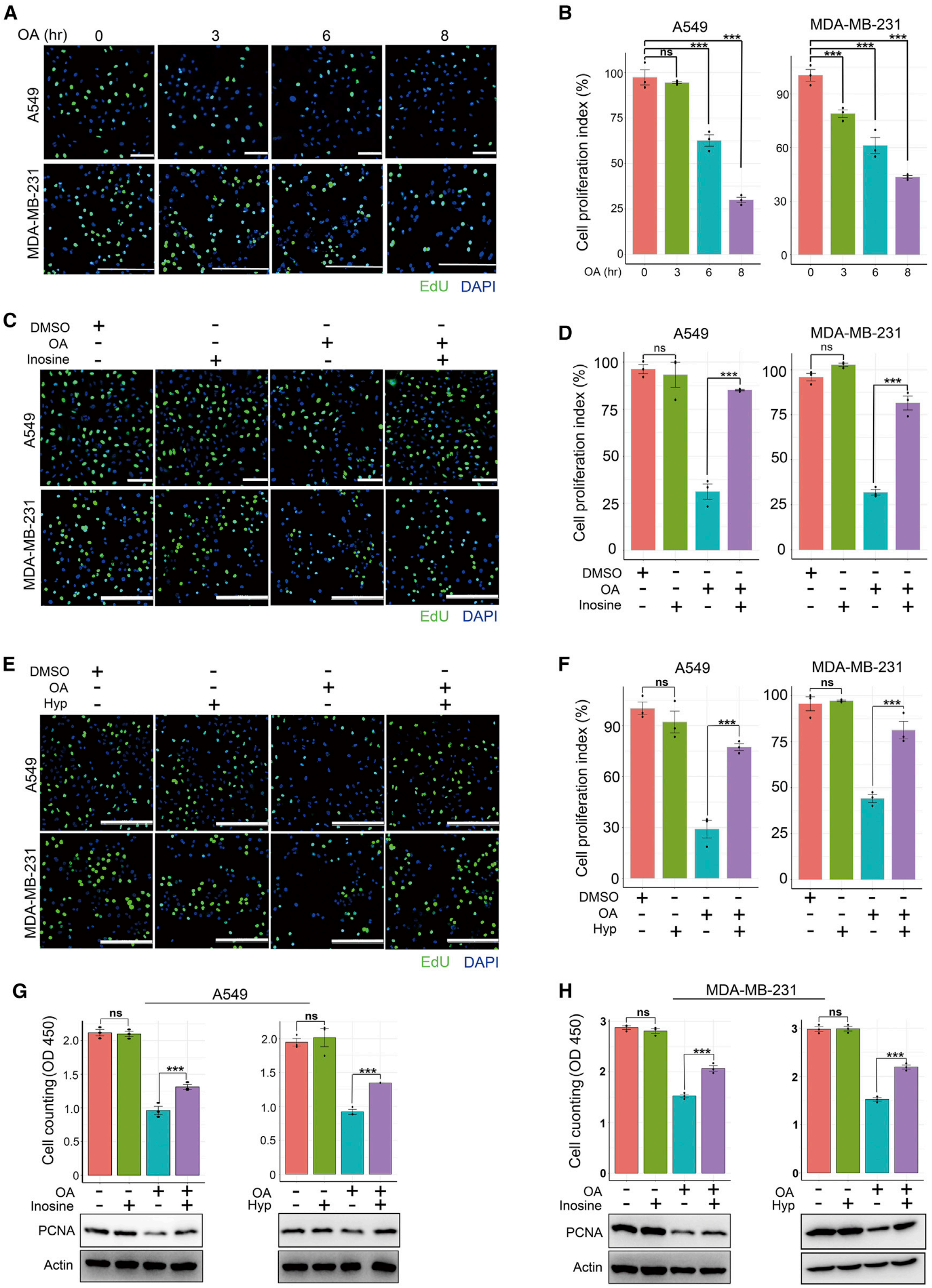

(legend on next page)

further confirm that OA accelerated autophagic flux in cancer cells, we adopted a previously reported approach.[32] During the time course of OA treatment, the autophagy inhibitor BAF dramatically enhanced the accumulation of LC3-II in A549 cells relative to the vehicle (Figure S5A), demonstrating that OA treatment expedited the autophagic flux of cancer cells. Unc-51-like autophagy activating kinase 1 (ULK1) is a key component of the autophagy apparatus that is activated by AMPK via phosphorylation of Ser555 or repressed by mTORC1 via phosphorylation of Ser757.[31] Our results showed that OA treatment significantly reduced phosphorylated ULK1 at Ser757 but did not influence phosphorylated ULK1 at Ser555 (Figure 6A). Thus, OA-induced AMPK activation may elicit autophagy by suppressing mTORC1. Therefore, we measured two well-known substrates of mTORC1, 4EBP1 and p70S6K, and found that OA treatment markedly decreased levels of phosphorylated 4EBP1 at Thr37/46 and phosphorylated p70S6K at Thr389 over time (Figure 6A; Figure S5B), demonstrating that OA inhibited mTORC1 activity. AMPK is a well-established negative upstream regulator of mTORC1.[33] Therefore, these results suggested that OA treatment activated the AMPK/mTORC1/autophagy pathway.

To determine whether OA-induced autophagy was required for HGPRT and 5′-NT degradation, we used two previously reported ULK1 inhibitors, ULK-101 and MRT68921,[34,35] to suppress autophagy in A549 cells. The results showed that both ULK1 inhibitors rapidly downregulated phosphorylated ATG14 at Ser29, a well-established substrate of ULK1,[36] reduced LC3-II isoform expression, and stabilized HGPRT and 5′-NT (Figure 6B). This result demonstrated that OA-induced autophagy contributed to HGPRT and 5′-NT degradation.

This form of autophagy is referred to as macroautophagy, which involves a multi-step process and formation of autophagosomes.[37] In addition to macroautophagy, the two other types of autophagy in cells include microautophagy and chaperone-mediated autophagy (CMA).[38,39] Thus, we ascertained whether these types of autophagy were activated by OA to contribute to HGPRT and 5′-NT degradation. TSG101 and LAMP-2A are two marker proteins for microautophagy and CMA, respectively.[39] OA treatment did not increase TSG101 and LAMP-2A expression in A549 cells (Figure S5C), indicating that OA did not activate microautophagy and CMA. 3-Methyladenine (3-MA) is another reported inhibitor of macroautophagy.[39] Treatment with 3-MA not only repressed LC3-II isoform expression but also curtailed HGPRT and 5′-NT degradation in A549 cells (Figure 6C). In addition, the blockade of microautophagy by knockdown of TSG101 did not alleviate OA-induced HGPRT and 5′-NT degradation, suggesting that microautophagy was not involved in OA-elicited proteolysis of these two metabolic enzymes (Figure S5D). Collectively, these results indicated that OA triggered macroautophagy, but not microautophagy and CMA, to initiate HGPRT and 5′-NT degradation.

Subsequently, we investigated whether OA-induced AMPK activation repressed mTORC1 activity, triggering macroautophagy and leading to lysosomal degradation of HGPRT and 5′-NT. AMPKα is a catalytic subunit of AMPK encoded by *PRKAA1*.[40] *PRKAA1* KO significantly downregulated total AMPK, phosphorylated AMPK at Thr172, and phosphorylated ACC at Ser79, indicating that *PRKAA1* KO markedly inhibited AMPK activity (Figure 6D). In OA-treated A549 cells, inhibition of AMPK activity by *PRKAA1* KO resulted in sustained activation of 4EBP1 and p70S6K, and inhibition of ULK1 (Figure 6D; Figure S5E). Consequently, OA-induced expression of LC3-II isoform was impeded and HGPRT and 5′-NT were stabilized (Figure 6D). Collectively, these results showed that OA-induced AMPK activation was required for the stimulation of the mTORC1/macroautophagy/lysosome pathway and degradation of HGPRT and 5′-NT.

Finally, we explored the upstream mechanism by which OA activated AMPK in cancer cells. OA treatment induces ROS generation in cancer cells.[41] ROS is a well-known upstream signal for AMPK activation.[42] Therefore, we hypothesized that OA treatment activated AMPK by inducing ROS generation. We observed that OA treatment for <8 h significantly upregulated ROS levels in A549 cells (Figure 6E). When OA-induced ROS were removed by the antioxidant *N*-acetyl-L-cysteine (NAC), phosphorylated AMPK at Thr172, phosphorylated ACC at Ser79, and LC3-II isoform were all restrained, while OA-induced HGPRT and 5′-NT degradation was blocked; thus, cancer cell growth was recovered (Figures 6E–6G). This result indicated that OA promoted ROS generation to activate the AMPK/macroautophagy/lysosome pathway, thereby degrading HGPRT and 5′-NT and impeding cancer cell growth. Energy deficit, as characterized by an increased ratio of ADP:ATP or AMP:ATP, is another well-known upstream signal that activates AMPK.[43] To ascertain whether OA caused an energy deficit, we measured the ADP:ATP ratio under OA treatment. The results showed that OA remarkably elevated the ADP:ATP ratio in A549 cells beginning at the 3rd hour of treatment (Figure S5F), indicating that an OA-induced energy deficit was potentially involved in activating AMPK. Notably, the ROS inhibitor NAC removed ∼81% of

Figure 3. PSP blockade is required for the anti-cancer activity of OA

(A and B) EdU (green) incorporation assay showing the impact of OA treatment (200 μM) on DNA replication of cancer cells. Cell nuclei were stained with 4′,6-diamidino-2-phenylindole (DAPI, blue). Scale bars: 100 μm, A549 cells; 250 μm, MDA-MB-231 cells. Quantitative analysis is shown in (B). (C–F) DNA replication arrest of cancer cells induced by OA treatment (200 μM) was restored by exogenous supplementation of inosine (80 μM) (C) or hypoxanthine (100 μM) (E) for 8 h. DNA replication was measured by EdU (green) incorporation, whereas cell nuclei were stained with DAPI (blue). Scale bars: 100 μm, A549 cells; 250 μm, MDA-MB-231 cells. Hyp, hypoxanthine. The quantitative results of (C) and (E) are shown in (D) and (F), respectively. (G and H) Curtailed cell proliferation and proliferating cell nuclear antigen (PCNA) expression induced by 200 μM OA treatment was recovered by exogenous supplementation of inosine (G) or hypoxanthine (H). Hyp, hypoxanthine. Cells were cultured for 72 h. Viable cell proliferation was measured with Cell Counting Kit-8 (CCK8). Error bars represent mean ± SEM. *p < 0.05, **p < 0.01, ***p < 0.001, Student's t test.

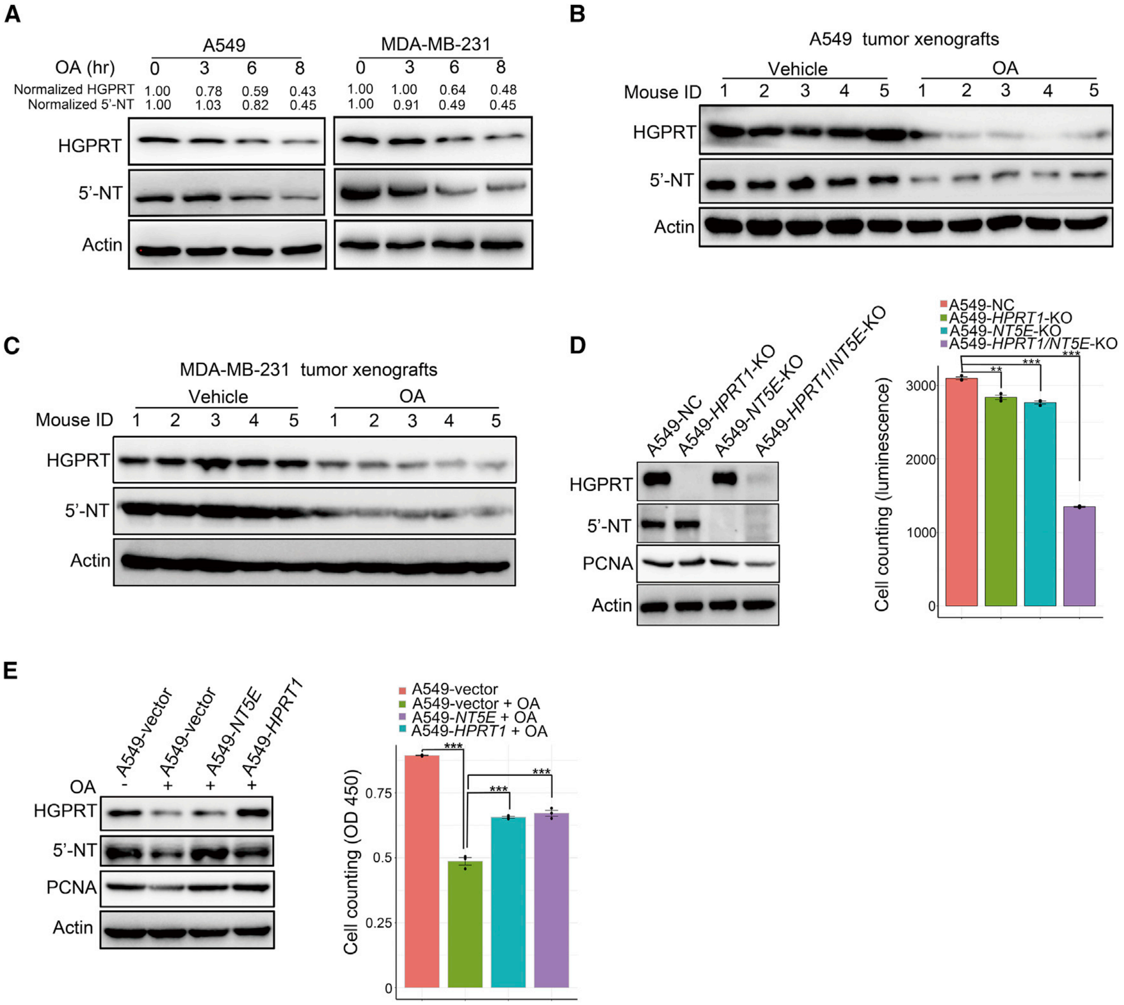

Figure 4. OA promptly downregulates two key metabolic enzymes in the PSP

(A) Western blot showing the time course of 200 μM OA treatment effects on protein levels of HGPRT and 5′-NT in A549 and MDA-MB-231 cells. (B and C) The influence of oral administration of OA on protein levels of HGPRT and 5′-NT in A549 (B) and MDA-MB-231 (C) tumor xenografts. OA was orally administered to the treatment group at 120 mg/kg/day until the end of the experiment. Twenty-two days after the subcutaneous injection of tumor cells, the tumor xenografts were resected for western blot assay. (D) The impact of individual and simultaneous deletion of *HPRT1* and *NT5E* on A549 cell proliferation. The cells were cultured for 72 h, and viable cells were counted with an ATPlite kit. (E) Individual overexpression of *HPRT1* and *NT5E* in A549 cells opposing proliferation arrest induced by 200 μM OA treatment. The cells were cultured for 72 h, and viable cells were counted by CCK-8. Error bars represent mean ± SEM. **p < 0.01, ***p < 0.001, Student's t test.

phosphorylated AMPK and 71% of phosphorylated ACC1 elicited by OA treatment for 8 h (Figure 6F), demonstrating that OA-induced ROS was a major upstream signal to stimulate AMPK in cancer cells.

Next, we determined which upstream factor was modulated by OA to lead to ROS production. Generally, there are two superoxide dismutases (SODs) in cells, SOD1 and SOD2, which are important for removing ROS.[44] We observed that OA administration rapidly suppressed the activity of total SODs but not SOD2, while overexpression of SOD1 remarkably reduced OA-induced ROS, enhanced HGPRT and 5′-NT stability, and reversed cell proliferation arrest elicited by OA (Figures 6H and 6I; Figure S5G). Furthermore, we applied a well-reported SOD1 inhibitor, LCS-1,[45] to determine whether the 40% reduction of SOD1 activity, as shown in Figure S5G, could elicit overt ROS accumulation in cancer cells. Treatment with 5 μM LCS-1 for 6 h resulted in ~40% decline in intracellular SOD1 activity in A549 cells, leading to a 1.7-fold increase in ROS levels in these cells (Figures S5H and S5I). In addition, we investigated whether *SOD1* knockdown mimicked the effects of OA. Downregulation of SOD1 expression by RNAi in A549 cells markedly stimulated AMPK and suppressed PCNA expression (Figure S5J), indicating that *SOD1* knockdown mimicked the effects of OA. Therefore, we concluded that OA selectively inactivated SOD1 and generated ROS to stimulate HGPRT and 5′-NT degradation via the AMPK/mTORC1/macroautophagy/lysosome pathway.

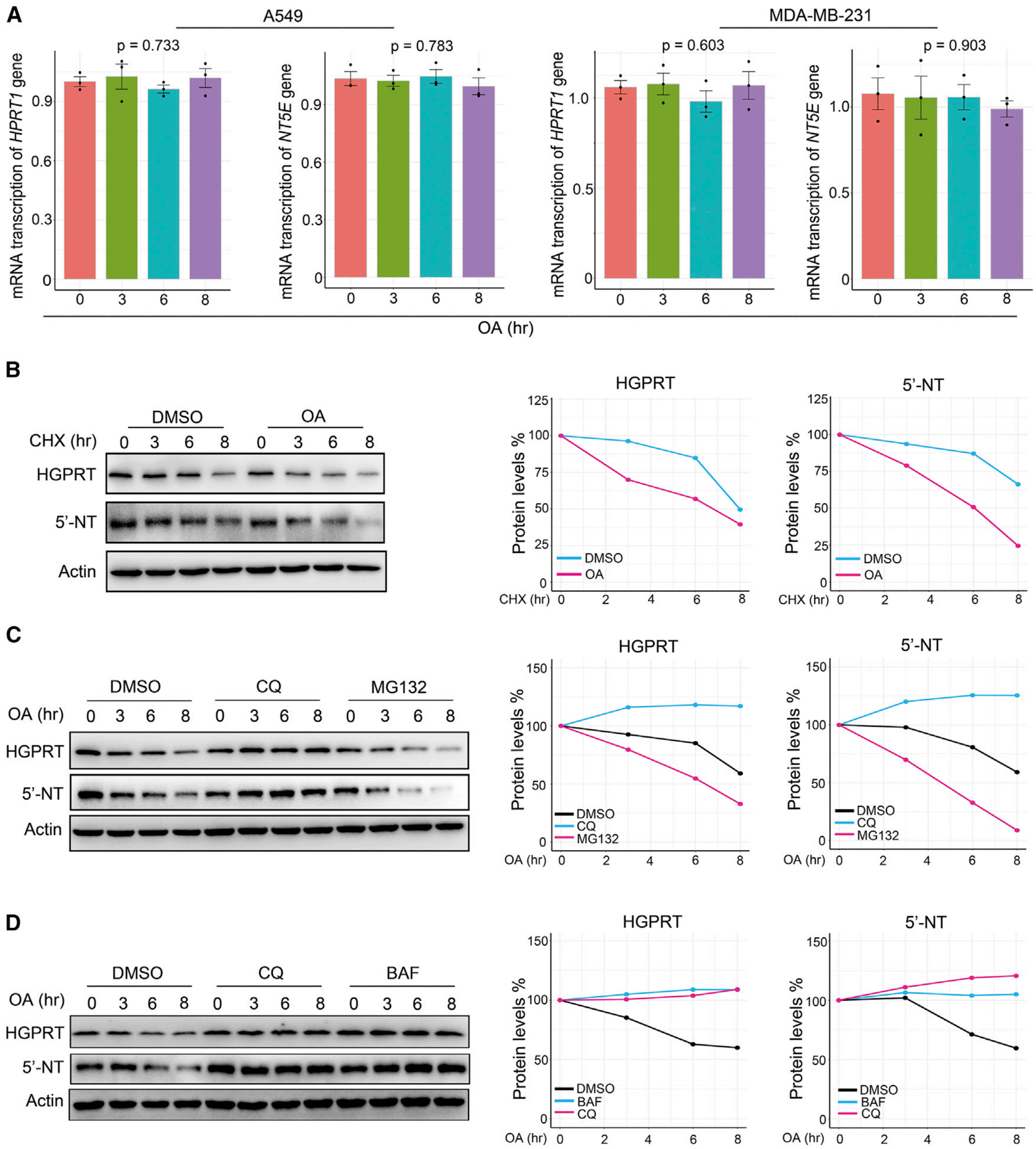

Figure 5. OA reduces HGPRT and 5′-NT in the PSP by activating lysosomal proteolysis

(A) Reverse transcription-quantitative polymerase chain reaction (RT-PCR) time course showing the impact of OA treatment (200 μM) on *HPRT1* and *NT5E* transcription in A549 and MDA-MB-231 cells. (B) The influence of OA (200 μM) on HGPRT and 5′-NT degradation when using cycloheximide (CHX) (50 μg/mL) to block protein synthesis in A549 cells. The curves on the right side of the western blot images indicate the quantification of protein levels. (C) The impact of lysosome inhibitor chloroquine (CQ) (0.02 μM) and the proteasome inhibitor MG132 (0.01 μM) on the degradation of HGPRT and 5′-NT induced by OA (200 μM) in A549 cells. The curves on the right side of the western blot images indicate the quantification of protein levels. (D) The influence of two lysosome inhibitors, CQ (0.02 μM) and bafilomycin A1 (BAF) (0.08 μM), on HGPRT and 5′-NT degradation induced by OA (200 μM) in A549 cells. The curves on the right side of the western blot images indicate the quantification of protein levels. Error bars represent mean ± SEM. *p < 0.05, **p < 0.01, ***p < 0.001, Student's t test.

The PSP is overactivated in human lung cancer and breast cancer with a negative linkage to patient survival

We next assessed the clinical relevance of the newly discovered downstream targets of OA and the PSP. First, we analyzed HGPRT and 5′-NT expression in the PSP in clinical tissue samples. In lung cancer patient cohort 1 (n = 5) (Table S1), the levels of both enzymes were elevated in cancerous tissues relative to their matched normal adjacent tissues (Figure 7A). In lung cancer patient cohort 2 (n = 34) (Table S2) and a breast

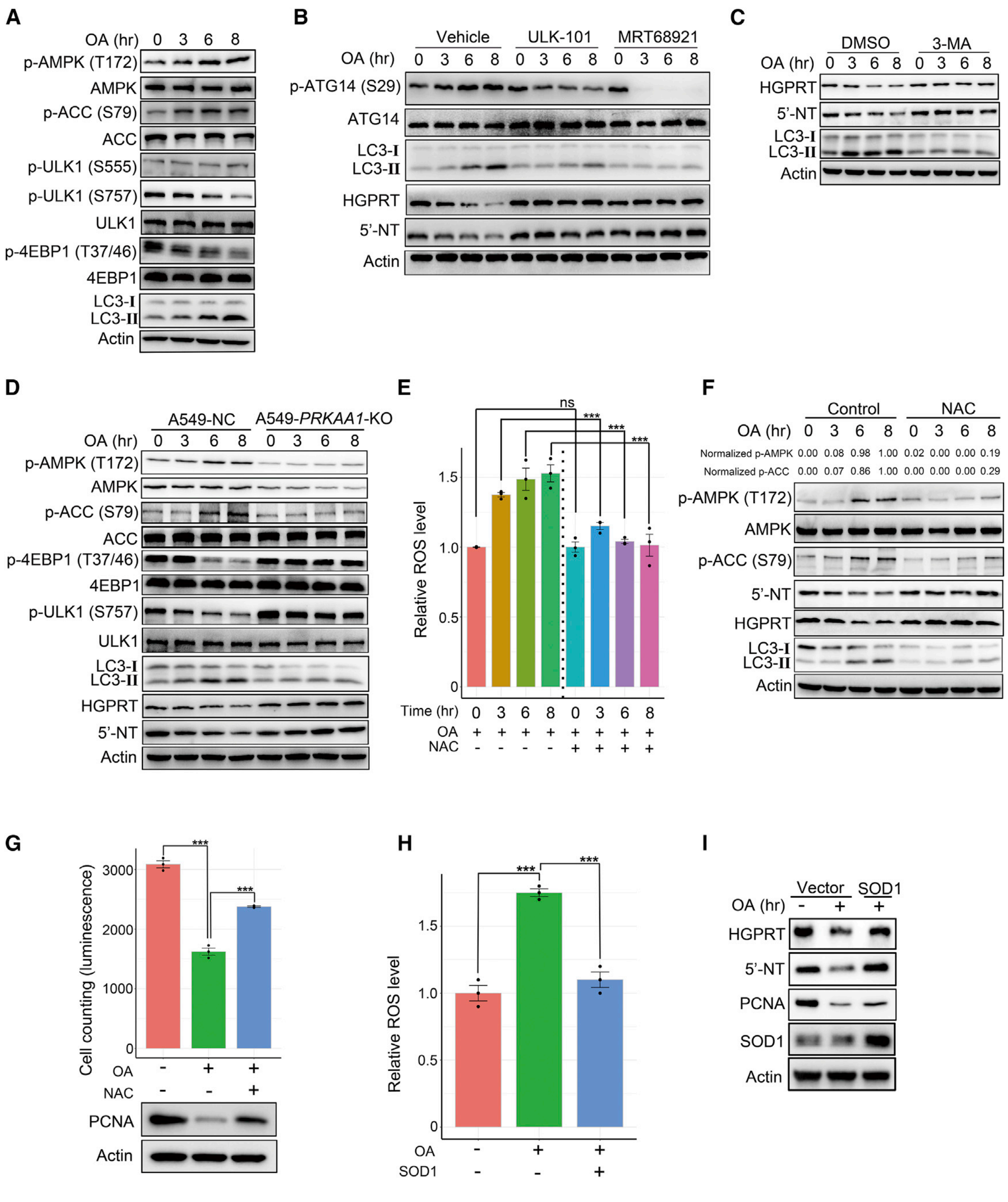

Figure 6. OA selectively inactivates SOD1 to degrade HGPRT and 5′-NT via the ROS/AMPK/mTORC1/macroautophagy/lysosome pathway

(A) Western blot showing the time course of the effects of OA treatment (200 μM) on the abundance of phospho-AMPK (Thr172), AMPK, phospho-ACC (Ser79), ACC, phospho-ULK1 (Ser555), phospho-ULK1 (Ser757), ULK1, phospho-4EBP1 (Thr37/46), 4EBP1, and LC3-I/II in A549 cells. (B) The effects of two ULK1 inhibitors, ULK-101 (1 μM) and MRT68921 (1 μM), on levels of phospho-ATG14 (Ser29), ATG14, LC3-I/II, HGPRT, and 5′-NT in OA-treated A549 cells. (C) Western blot showing the influence of 3-MA (1 mM) on HGPRT, 5′-NT, and LC3-I/II expression in OA-treated A549 cells. (D) Western blot showing the time course of the effects of 200 μM OA treatment on the expression of phospho-AMPK (Thr172), AMPK, phospho-ACC (Ser79), ACC, phospho-ULK1 (Ser757), ULK1, phospho-4EBP1 (Thr37/46), 4EBP1, LC3-I/II, HGPRT, and 5′-NT in A549 cells with and without *PRKAA1* KO. (E) Measurement of reactive oxygen species (ROS) generation induced by OA (200 μM) and ROS removal following *N*-acetyl-L-cysteine (NAC) (5 mM) treatment in A549 cells. (F) Time course of the reversion of phospho-AMPK (Thr172), phospho-ACC (Ser79), ACC, HGPRT, 5′-NT, and LC3-I/II in OA-treated A549 cells after NAC (5 mM). The values of phospho-AMPK (Thr172) were normalized as follows: the raw abundance of phospho-AMPK (Thr172) of each lane was first normalized by the corresponding actin, and the actin-normalized proteins of all lanes were then further normalized by the actin-normalized protein on the

(legend continued on next page)

cancer patient cohort (n = 20) (Table S3), the levels of these enzymes were also significantly upregulated in cancerous tissues (Figures 7B and 7C). Notably, the enzyme activities of HGPRT and 5′-NT were markedly upregulated in cancerous tissues relative to matched normal adjacent tissues derived from lung cancer patient cohort 1 (Figure 7D). Second, we analyzed the purine metabolites of the PSP in clinical tissue samples. In lung cancer patient cohort 3 (n = 34) (Table S4), five metabolites in the PSP were significantly elevated in cancer tissues (Figure 7E). Collectively, these results indicated PSP overactivation in human lung and breast cancers.

Subsequently, we examined whether the PSP affected cancer mortality by investigating the association between *HPRT1* or *NT5E* expression and the overall survival rate of patients with cancer in the public TCGA and GTEx databases with the online bioinformatics analysis tool GEPIA2 (http://gepia2.cancer-pku.cn/).[46] We found that high *HPRT1* or *NT5E* expression was closely associated with inferior overall survival in patients with lung or breast cancers (Figures 7F and 7G).

In conclusion, the present study uncovered a new mechanism for the herbal compound OA to impair cancer cell growth by blocking the PSP via the degradation of key metabolic enzymes in this pathway through the SOD1/ROS/AMPK/mTORC1/macroautophagy/lysosome pathway (Figure 7H).

DISCUSSION

This study thoroughly evaluated the metabolic targets of OA, an important compound in natural plants with anti-cancer applications, using a metabolomic approach. The results of this study revealed two novel findings. First, we determined that the PSP is a key metabolic target through which OA demonstrates anti-cancer activity. Second, we elucidated the molecular mechanism by which OA blocks the PSP by selectively inactivating SOD1 and degrading PSP enzymes, including HGPRT and 5′-NT, via the ROS/AMPK/mTORC1/macroautophagy/lysosome pathway. Notably, OA treatment also inhibited other metabolic pathways, such as glucose-alanine cycle, glycolysis, and galactose metabolism (Figure 2C). These metabolic pathways are important for cancer cell growth.[20,47] Therefore, it is reasonable to speculate that OA-stimulated autophagy could also degrade the metabolic enzymes to prevent these pathways and suppress cancer cell malignancy. Taken together, these findings not only ascertained the crucial metabolic target of OA but also deepened our understanding of the molecular mechanism by which this natural compound modulates cancer metabolism.

In cancer cells, purine *de novo* synthesis, and not salvage synthesis, is considered a fundamental pathway to replenish the purine pool.[17,18,48,49] However, our study revealed that the PSP is essential for the homeostasis of the purine pool and plays a crucial role in cancer cells. Two lines of evidence support this conclusion. OA treatment dramatically reduced the levels of PSP metabolites and PSP enzymes, as well as the activity of PSP enzymes in cancer cells, whereas exogenous supplementation of PSP metabolites significantly restored DNA replication and cancer cell growth impeded by OA. Furthermore, immunoblot, immunohistochemistry (IHC), enzyme activity, and metabolomic investigations of our patient cohorts showed overactivated PSP activity in human lung and breast cancers, while bioinformatics analysis of public databases showed that high expression of PSP enzymes was linked to poor patient survival. Notably, the overexpression of PSP enzymes including HGPRT and 5′-NT in both cancer cell lines and primary cancer cells of patients has been reported recently.[50,51] As the PSP is a key downstream metabolic target of OA, as shown by the current study, it is reasonable to speculate that OA would show efficacy in cancer cells with high PSP activity. Which types of cancer are sensitive to OA treatment based on the PSP activity in human cancers requires further investigation.

AMPK is a key player in the elegant system that accurately modulates cellular metabolism based on nutrient availability.[33] Although AMPK activation by OA has been reported,[9] the underlying molecular mechanism was unknown. The results of the current study demonstrated that OA selectively inactivated SOD1, an important regulator of redox homeostasis,[52] thereby upregulating ROS and stimulating AMPK. Previous studies on the mechanisms by which AMPK regulates cellular metabolism mainly focused on its effects on the activity of various signal proteins (mTORC1 and TSC2) and metabolic enzymes (ACC1 and ACC2) via phosphorylation of these protein substrates.[33] The results of the present study showed that AMPK activated by OA dramatically expedited lysosomal degradation of the metabolic enzymes in the PSP by inhibiting mTORC1 activity and stimulating macroautophagy. Therefore, this study elucidated the mechanism by which OA activates AMPK and revealed a new mechanism by which AMPK modulates cellular metabolism by promoting the lysosomal degradation of metabolic enzymes.

Finally, the translational potential of OA for cancer therapy highlighted in the present study should be noted. The efficacy of OA administration on lung cancer A549 cells and breast cancer MDA-MB-231 cells *in vitro* and *in vivo* demonstrated the potential of this natural compound in cancers with high PSP activity. In addition, the *in vivo* use of this compound showed negligible side effects, indicating its safety. Furthermore, metabolic inhibitors exhibit obvious synergistic effects with conventional chemotherapeutics.[14,20,53] It would be interesting to test in the future whether a combination of OA and conventional chemotherapeutic drugs can give rise to synergistic anti-cancer efficacy and further refine patient outcomes.

fourth lane (control group treated by OA for 8 h). Normalized phospho-ACC (Ser79) was acquired by the same computation approach. (G) Restoration of impaired cell proliferation and PCNA expression by NAC with OA treatment (200 μM). Viable cell proliferation was measured with an ATPlite kit. (H) Measurement of ROS generation in OA-treated A549 cells with and without SOD1 overexpression. (I) Under OA treatment, SOD1 overexpression enhanced HGPRT and 5′-NT stability and restored PCNA expression. Error bars represent mean ± SEM. *p < 0.05, **p < 0.01, ***p < 0.001, Student's t test. Ns, no significance.

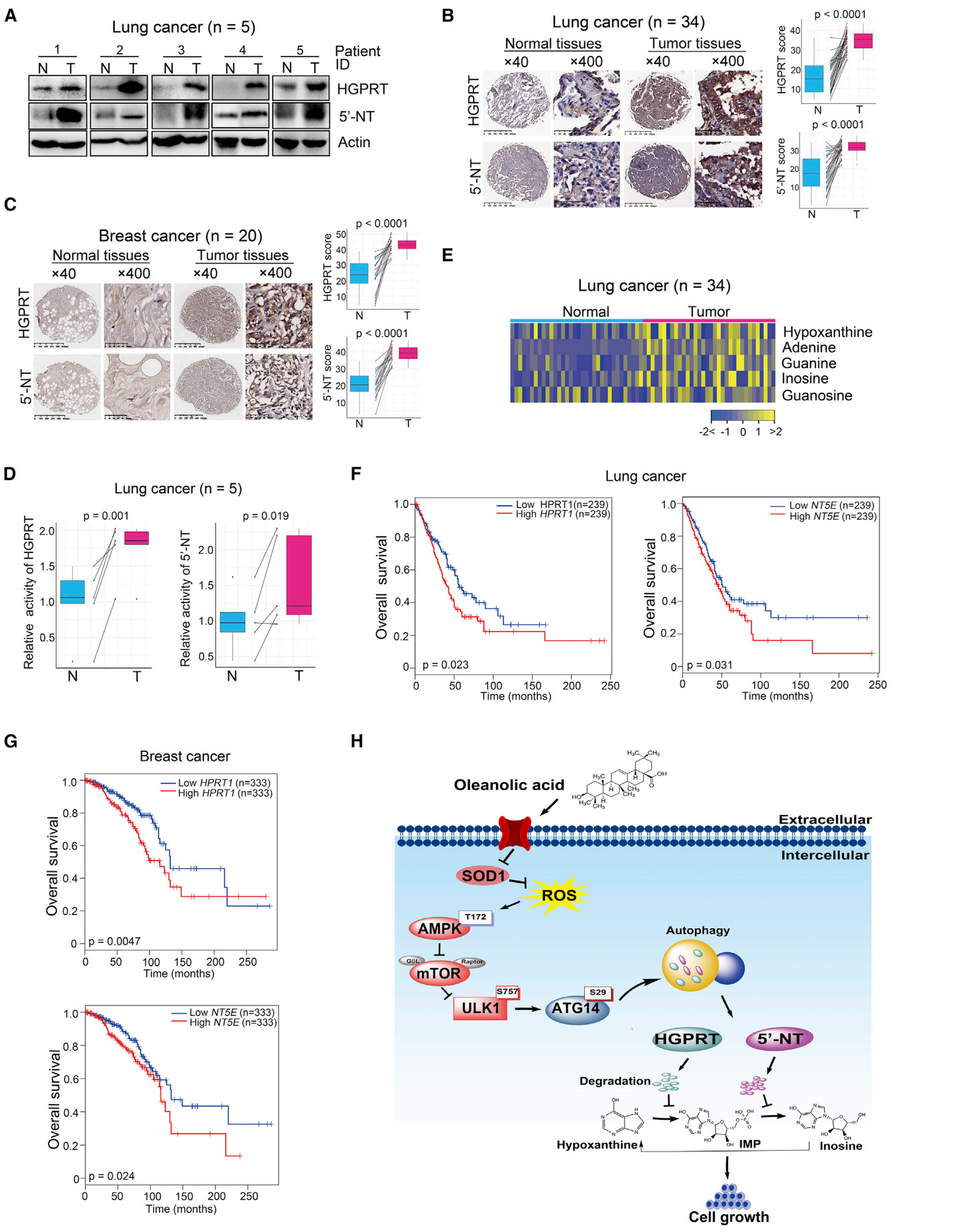

(legend on next page)

MATERIALS AND METHODS

Cell culture and reagents

The human lung cancer cell line A549 was obtained from the National Cancer Institute, whereas the human breast cancer cell line MDA-MB-231 was purchased from American Type Culture Collection (ATCC, Manassas, VA, USA). Cell identities of these two cell lines were authenticated by short tandem repeat profiling. Both cell lines were maintained in Dulbecco's modified Eagle's medium (DMEM; Thermo Scientific, Waltham, MA, USA) supplemented with 10% fetal bovine serum (FBS; Thermo Scientific, Waltham, MA, USA). Cells were cultured at 37°C and 5% CO_2 in a cell incubator. The reagents used in this study included OA (Sigma-Aldrich, St. Louis, MO, USA), dimethyl sulfoxide (DMSO; Sigma-Aldrich, St. Louis, MO, USA), hypoxanthine (MedChemExpress, Monmouth Junction, NJ, USA), inosine (MedChemExpress, Monmouth Junction, NJ, USA), cycloheximide (Sigma-Aldrich, St. Louis, MO, USA), MG132 (MedChemExpress, Monmouth Junction, NJ, USA), CQ (Cell Signaling Technology, Boston, MA, USA), BAF (Cell Signaling Technology, Boston, MA, USA), ULK-101 (MedChemExpress, Monmouth Junction, NJ, USA), MRT68921 (MedChemExpress, Monmouth Junction, NJ, USA), IMP (MedChemExpress, Monmouth Junction, NJ, USA), 3-MA (MedChemExpress, Monmouth Junction, NJ, USA), NAC (MedChemExpress, Monmouth Junction, NJ, USA), SOD1 inhibitor LCS-1 (Sigma-Aldrich, St. Louis, MO, USA), and NP40 buffer (Beyotime, Shanghai, China).

Cell viability and cell colony formation assays

For cell viability analysis, cells were seeded into 96-well plates at a density of 3,000/well for A549 cells and 2,000/well for MDA-MB-231 cells in medium prepared with DMEM, 10% FBS, and reagents indicated in figures or figure legends. Cell viability was analyzed with a Cell Counting Kit-8 (CCK-8, Dojindo Laboratories, Kumamoto-ken, Japan) or ATPlite (PerkinElmer, Waltham, MA, USA) according to the manufacturer's recommendation.

For cell colony formation assay, cells were seeded into 6-well plates at a density of 500/well. Distinct concentrations of OA were added into cell medium. Cell colonies were stained with 0.05% (v/v) crystal violet (Sigma-Aldrich, St. Louis, MO, USA) and counted with ImageJ software (Version 1.8.0_112, https://imagej.nih.gov/ij/).

Assessment of the therapeutic efficacy of OA on tumor xenograft growth *in vivo*

A549 (5×10^6) or MDA-MB-231 (3×10^6) cells were subcutaneously injected into the right hind flanks of 8-week-old female BALB/c-nude mice (Shanghai SLAC Laboratory Animal, Shanghai, China). After 8 days of subcutaneous injection, mice were randomly divided into the treatment group and the vehicle group. OA solution was orally administered to the treatment group with a dosage of 120 mg/kg/day as described previously.[9] The same volume of physiological saline was given to the vehicle group. OA was administered until the end of the experiment. The tumor length and width were measured every 3 days by caliper, and tumor sizes were calculated with a formula of $0.5 \times \text{length} \times \text{width}^2$. After 22 days from subcutaneous injection of tumor cells, tumor xenografts were resected for imaging and weighing. Subsequently, tumor xenografts were flash-frozen and stored in liquid nitrogen until western blot and metabolomic assay. Mouse studies were performed in specific pathogen-free (SPF) facilities with approval of the Institutional Animal Care and Use Committee of Fudan University (SYXK(Hu)2014-0029).

Lactate production assay

A549 cells were seeded into 96-well plates at a density of 1×10^4/well in glucose-free DMEM with 10% dialyzed fetal bovine serum (dFBS; Thermo Scientific, Waltham, MA, USA) and 6 mM glucose. Spent media at distinct time points were collected for examination of lactate production with an Amplite Colorimetric L-Lactate Assay kit (AAT Bioquest, Sunnyvale, CA, USA) according to the manufacturer's protocol.

Enzyme activity assays of HGPRT and 5′-NT in cell samples and clinical tissue specimens

For cells, A549 cells were seeded into 6-well plates at a density of 1.5×10^5/well and treated with 200 μM OA for 8 h. After removal of the medium, cells were harvested into 1.5-mL tubes and 200 μL of lysis solution was added into each tube to acquire cell lysates. For tissues, ~15 mg of each histological tissue of each patient was weighed and put into a 2-mL tube, and 100 μL of lysis solution was then added into each tube to obtain cell lysates. The enzyme activity of HGPRT of each sample was measured with a PicoProbe Hypoxanthine Phosphoribosyl Transferase Activity Assay Kit (Fluorometric) (BioVision,

Figure 7. The PSP is overactivated in human lung cancer and breast cancer, with a negative linkage to patient survival

(A) Western blot showing HGPRT and 5′-NT expression in tumorous and paired normal adjacent tissues from patients with lung adenocarcinoma (n = 5). (B) Representative immunohistochemistry stained images of lung tissue microarrays using HGPRT and 5′-NT antibodies from samples obtained from patients with lung cancer (n = 34). Scale bars for ×40 images, 625 μm; scale bars for ×400 images, 50 μm. The midlines of the boxplots represent the median values of the data, with the upper and lower limits of the box indicating the third and first quartiles and the whiskers of the boxplot up to 1.5 times the interquartile ranges. The p values were calculated with non-parametric Wilcoxon rank-sum tests. N, paired normal adjacent lung tissues; T, tumorous lung tissues. (C) Representative immunohistochemistry stained images of lung tissue microarrays using HGPRT and 5′-NT antibodies in samples from patients with breast cancer (n = 20). Scale bars for ×40 images, 625 μm; scale bars for ×400 images, 50 μm. The midlines of the boxplots represent the median value of the data, with the upper and lower limits of the box indicating the third and first quartiles and the whiskers up to 1.5 times the interquartile ranges. The p values were calculated with non-parametric Wilcoxon rank-sum tests. N, paired normal adjacent breast tissues; T, tumorous breast tissues. (D) Enzyme activity of HGPRT and 5′-NT in tumorous and paired normal adjacent tissues derived from patients with lung adenocarcinoma (n = 5). The p values were acquired by Student's t test. (E) Differentially expressed PSP metabolites between tumorous and normal adjacent lung tissues from patients with lung cancer (n = 34). (F and G) Kaplan-Meier curves showing the association of expression of *HPRT1* or *NT5E* and overall survival of patients with lung or breast cancer from the public TCGA and GTEx databases. The p values were gained by log-rank test. (H) A model proposed by the current study for the herbal compound OA. OA impaired cancer cell growth by blocking the PSP by degrading HGPRT and 5′-NT in this pathway through the SOD1/ROS/AMPK/mTORC1/autophagy/lysosome pathway.

Milpitas, CA, USA), and the enzyme activity of the 5′-NT of each sample was assessed with a 5′-Nucleotidase (CD73) Activity Kit (Colorimetric) (Abcam, Cambridge, UK). Enzyme activity assays were conducted according to the manufacturers' instructions.

Metabolomic profiling of cell and tissue samples

For each cell sample, 1×10^7 cells were harvested and extracted to acquire metabolites. For each tissue sample, ~20 mg of tissue was weighed and homogenized for metabolite extraction. Subsequently, GC-TOFMS (LECO, St. Joseph, MI, USA) was used for metabolite measurement. Metabolomic assay was carried out by Metabo-Profile (Shanghai, China), using previously published methods.[20,54] The metabolites were identified by comparison with the internal library built with standard reference compounds.

Knockdown of *HPRT1*, *NT5E*, and *PRKAA1* by means of CRISPR-Cas9 approach

We downregulated *HPRT1*, *NT5E*, and *PRKAA1* in cancer cells with CRISPR-Cas9 technology as described previously.[55] In brief, nontarget control (NC) guide RNA (gRNA) duplex (Forward 5′-AAG AAGAATTGGGGATGATG-3′; Reverse 5′-CATCATCCCCAATTC TTCTT-3′), gRNA duplex targeting *HPRT1* encoding HGPRT (Forward 5′-GAGCTGCTCACCACGACGCC-3′; Reverse 5′-GGCGTCG TGGTGAGCAGCTC-3′), gRNA duplex targeting *NT5E* encoding 5′-NT (Forward 5′-TTACCATGGCATCGTAGCGC-3′; Reverse 5′-G CGCTACGATGCCATGGTAA-3′), and gRNA duplex targeting *PRKAA1* encoding AMPKα (Forward 5′-AGTAAAAACAGGCTCC ACGA-3′; Reverse 5′-TCGTGGAGCCTGTTTTTACT-3′) were inserted into the lenti-Guide-CRISPR-v2-puro vector, respectively. The lentivirus was produced as follows: lenti-Guide-CRISPR-v2-puro vector containing NC gRNA or lenti-Guide-CRISPR-v2-puro vector containing target gRNA was co-transfected with psPAX2 and pMD2.G plasmids into HEK293T cells with a Lipofectamine 3000 Transfection Reagent (Thermo Fisher Scientific, Waltham, MA, USA) according to the manufacturer's instruction. After 48 h of incubation, lentivirus-containing supernatants were collected and filtered (0.45-μm filter) to remove cells. Subsequently, cancer cells were infected with lentivirus with NC gRNA or lentivirus with target gRNA in the presence of 8 μg/mL polybrene. Infected cells were selected with puromycin for 48 h.

Overexpression of *HPRT1* and *NT5E*

We overexpressed *HPRT1* or *NT5E* in cancer cells with CRISPR-Cas9-SAM technology as described previously.[56] First, lenti-MPH-v2 vector was co-transfected with psPAX2 and pMD2.G plasmids into HEK293T cells with a Lipofectamine 3000 Transfection Reagent (Thermo Fisher Scientific, Waltham, MA, USA) according to the manufacturer's instruction. After 48 h of incubation, lentivirus-containing supernatants were collected and filtered (0.45-μm filter) to remove cells. Subsequently, cancer cells were infected with the lentivirus lenti-MPH-v2 in the presence of 8 μg/mL polybrene. Infected cells, which were selected with hygromycin for 7 days, were used for the following lentivirus infection.

Second, the lentivirus lenti-SAM-v2-puro was constructed for enforced expression of targeted genes. NC gRNA duplex (Forward 5′-A AGAAGAATTGGGGATGATG-3′; Reverse 5′-CATCATCCCCAA TTCTTCTT-3′), gRNA duplex targeting *HPRT1* encoding HGPRT (Forward 5′-CAGGCTCACTAGGTAGCCGT-3′; Reverse 5′-ACG GCTACCTAGTGAGCCTG-3′), and gRNA duplex targeting *NT5E* encoding 5′-NT (Forward 5′-TCGTGCGTTCTCAACCCAAC-3′; Reverse 5′-GTTGGGTTGAGAACGCACGA-3′) were inserted into the lenti-SAM-v2-puro vector, respectively. The lentivirus was produced as follows: lenti-SAM-v2-puro vector containing NC gRNA or lenti-SAM-v2-puro vector containing target gRNA was co-transfected with psPAX2 and pMD2.G plasmids into HEK293T cells with a Lipofectamine 3000 Transfection Reagent (Thermo Fisher Scientific, Waltham, MA, USA) according to the manufacturer's recommendation. Subsequently, cancer cells were infected with lentivirus with NC gRNA or lentivirus with target gRNA in the presence of 8 μg/mL polybrene. Infected cells were selected with puromycin for 48 h.

Measurement of DNA replication in cells with 5-ethynyl-2′-deoxyuridine incorporation assay

Cells were seeded into 6-well plates at a density of 3×10^5/well for A549 cells and 3×10^5/well for MDA-MB-231 cells in medium prepared with DMEM, 10% FBS, and reagents indicated in figures or figure legends. At the time points designated, cells were labeled with EdU (Beyotime, Shanghai, China) at a concentration of 10 μM at 37°C for 2 h. After removal of the medium, cells were fixed in 4% paraformaldehyde for 15 min and then permeabilized with 0.03% Triton X-100 for 10 min at room temperature. EdU incorporation was assessed with a Beyoclick EdU Cell Proliferation Kit with Alexa Fluor 488 (Beyotime, Shanghai, China) according to the manufacturer's instructions.

Quantitative RT-PCR

The transcription of metabolic genes involved in PSP was measured by quantitative RT-PCR (qRT-PCR). Actin was used as the internal reference. The assay was carried out with a SYBR Premix Ex Taq (Tli RNaseH Plus) Kit (Takara, Ostu, Japan) on an Applied Biosystems Q5 PCR machine (Applied Biosystems, Foster City, CA, USA). All primers are listed in Table S5.

Western blot and antibodies

Cells cultured *in vitro* were digested with 0.25% trypsin and lysed with RIPA buffer (Sigma-Aldrich, St. Louis, MO, USA) containing 1% protease inhibitor cocktail (v/v; Sigma-Aldrich, St. Louis, MO, USA) on ice. For tumor xenografts, tissues were cut into small pieces and homogenized in RIPA buffer containing 1% protease inhibitor cocktail on ice. Supernatants of cell/tissue lysates containing total proteins were acquired by centrifugation at 12,000 × *g* for 10 min, and their concentrations were determined by use of a BCA Assay Kit (Thermo Scientific, Waltham, MA, USA). Protein extracts were denatured by addition of sample loading buffer (Bio-Rad, Hercules, CA, USA) followed by boiling for 10 min, resolved by SDS-PAGE, and then transferred to polyvinylidene fluoride (PVDF) membranes. After incubation with primary antibodies overnight at 4°C, the membranes were washed and then

incubated with secondary antibodies conjugated with immunoglobulin G (IgG)-horseradish peroxidase (HRP) (Cell Signaling Technology, Boston, MA, USA). Primary antibodies against human PCNA (Cell Signaling Technology, Boston, MA, USA), human HGPRT (ab10479, Abcam, Cambridge, UK), human 5′-NT (Cell Signaling Technology, Boston, MA, USA), AMPK (Cell Signaling Technology, Boston, MA, USA), LAMP-2A (Abcam, Cambridge, UK), phospho-AMPK (Thr172) (Cell Signaling Technology, Boston, MA, USA), LC3-I/II (Cell Signaling Technology, Boston, MA, USA), ULK1 (Cell Signaling Technology, Boston, MA, USA), phospho-ULK1 (Ser555) (Cell Signaling Technology, Boston, MA, USA), phospho-ULK1 (Ser757) (Cell Signaling Technology, Boston, MA, USA), phospho-4EBP1 (Thr37/46) (Cell Signaling Technology, Boston, MA, USA), 4EBP1 (Cell Signaling Technology, Boston, MA, USA), ATG14 (Cell Signaling Technology, MA, Boston, USA), phospho-ATG14 (Ser29) (Cell Signaling Technology, Boston, MA, USA), ACC (Cell Signaling Technology, Boston, MA, USA), phospho-ACC (Ser79) (Cell Signaling Technology, Boston, MA, USA), phospho-p70S6K (Thr389) (Cell Signaling Technology, Boston, MA, USA), p70S6K (Cell Signaling Technology, Boston, MA, USA), SOD1 (Proteintech, Wuhan, China), GAPDH (Cell Signaling Technology, Boston, MA, USA), and Actin (Cell Signaling Technology, Boston, MA, USA) were enrolled in this study.

ADP/ATP ratio measurement

A549 cells were seeded into 6-cm dishes at a density of 5×10^5/dish and then treated with 200 μM OA for 0, 3, or 6 h. After removal of the medium, cells of each dish were harvested into a 1.5-mL tube and 100 μL of lysis solution was added into each tube to generate cell lysates. The ADP/ATP ratio of cells of each dish was analyzed with an ADP/ATP Ratio Assay Kit (bioluminescent) (Sigma-Aldrich, St. Louis, MO, USA) according to the manufacturer's protocol.

Measurement of reactive oxygen species

Generation of ROS was measured by the reagent 2′,7′-dichlorofluorescein diacetate (DCFH-DA; Sigma-Aldrich, St. Louis, MO, USA) as described previously.[57] Briefly, A549 cells (1×10^4/well) treated with 200 μM OA at distinct time points were incubated in culture medium containing 10 μM DCFH-DA for 30 min at 37°C, washed with serum-free medium three times, and analyzed with a fluorescence spectrophotometer (excitation 488 nm, emission 525 nm).

Activity assays of total superoxide dismutases and superoxide dismutase 2

A549 cells were seeded into 6-well plates at a density of 2×10^5/well and treated with 200 μM OA for 0, 3, or 6 h or treated with 5 μM LCS-1 for 6 h. After removal of the medium by centrifugation, cells of each well were harvested into a 1.5-mL tube and 200 μL of lysis solution was added into each tube to acquire cell lysates. The total SOD activity of each sample was assessed with a Total Superoxide Dismutase Assay Kit with WST-8 (Beyotime, Shanghai, China), and the SOD2 activity was measured with a Cu/Zn-SOD and Mn-SOD Assay Kit with WST-8 (Beyotime, Shanghai, China).

Overexpression of superoxide dismutase 1

Superoxide dismutase 1 (*SOD1*) sequence was cloned with a pair of primers (Forward 5′-CAGGTGCCACTCCCAGGTCCAAG-3′; Reverse 5′-GGCAACTAGAAGGCACAGTCGAGG-3′) and inserted into the pCMV3-Flag vector (Sino Biological, Beijing, China). The control vector pCMV3-Flag and the recombinant vector pCMV3-Flag-*SOD1* were utilized for cell transfection according to the manufacturer's instructions. The transfection was carried out with a Lipofectamine 3000 Transfection Reagent (Thermo Scientific, Waltham, MA, USA) according to the manufacturer's protocol.

Human cancer tissue acquirement and immunohistochemistry staining

We enrolled three lung cancer patient cohorts and one breast cancer patient cohort in this study. Lung cancer patient cohort 1 (n = 5) (Table S1) was from Nantong Cancer Hospital. Lung cancer patient cohort 2 (n = 34) (Table S2) and breast cancer patient cohort (n = 20) (Table S3) were from Longhua Hospital. Lung cancer patient cohort 3 (n = 34) (Table S4) was from Xinhua Hospital. All participants provided informed written consent in accordance with the regulations of the Institutional Review Board of each hospital in agreement with the Declaration of Helsinki (Approval number 2019-022). All of these enrolled patients had received no prior treatment for their disease. Paired adjacent benign and tumorous tissues of these patients were collected when they underwent surgery. Specimens from lung cancer patient cohort 1, cohort 2, and cohort 3 were used for western blot measurement, IHC staining, and metabolomic assay, respectively, whereas specimens from the breast cancer patient cohort were only used for IHC staining.

Tissue samples from lung cancer patient cohort 2 and the breast cancer patient cohort were used to construct a tissue microarray as described previously.[14,58] Tissue microarrays were stained with antibody against HGPRT, 5'-NT, or nonspecific IgG as a negative control. The tissue sections were quantitatively scored based on the percentage of positive cells and staining intensity as described previously.[59] The mean percentage of positive cells was computed in five areas of a given sample at a magnification of ×400 and scored from 0 to 100%. The staining intensity was scored as 0 for negative, 1 for weak, 2 for moderate, and 3 for strong. The proportion and intensity scores were then combined to obtain a weighted staining score for each case, ranging from 0 (0% of cells stained) to 3 (100% of cells stained).

RNA interference

The following small interfering RNAs (siRNAs) were synthesized by Biotend, Shanghai, China: si-nontarget control (siNC): sense sequence 5′-UUCUCCGAACGUGUCACGU-3′, antisense sequence 5′-UUCUCCGAACGUGUCACGU-3′; siTSG101: sense sequence 5′-CGUGAAACUGUCAAUGUUA-3′, antisense sequence 5′-UAACAUUGACAGUUUCACG-3′; siSOD1-1#: sense sequence 5′-CGAGCAGAAGGAAAGUAAU-3′, antisense sequence 5′-AUUACUUUCCUUCUGCUCG-3′; siSOD1-2#: sense sequence 5′-GGUGGAAAUGAAGAAAGUA-3′, antisense sequence 5′-UACUUUCUUCAUUUCCACC-3′. Oligonucleotides were resuspended according to the supplier's

instructions. The siRNA transfection was carried out with a Lipofectamine RNAiMAX Transfection Reagent (Thermo Scientific, Waltham, MA, USA) according to the manufacturer's protocol.

Statistical analysis

Statistical analysis was performed with R software (R version 3.4.3, https://www.r-project.org/). For multivariate analysis of metabolomics data, a principal component analysis model was fitted to capture the metabolic signatures of different groups with an R package of mixOmics.[60] Differential abundance scores of metabolic pathways were computed to evaluate the impact of OA treatment on the activity of metabolic pathways by using an algorithm reported previously.[24]

Significant differences between two groups were computed with Student's t test or nonparametric Wilcoxon rank-sum test. Survival analysis was performed by means of Kaplan-Meier method followed by log-rank test.

SUPPLEMENTAL INFORMATION

Supplemental information can be found online at https://doi.org/10.1016/j.omto.2021.08.013.

ACKNOWLEDGMENTS

This work was supported by National Natural Science Foundation of China (31970708, 81770147, 81625018, 81572340, 81802891, 31970708, 3217050266, 82002953), the National Scientific and Technological Major Special Project of China (2019ZX09201004-002-013), National Key R&D Program of China (2016YFA0501800), National Thirteenth Five-Year Science and Technology Major Special Project for New Drug Innovation and Development (2017ZX09304 001), Research fund of Shanghai Municipal Commission of Health (20174Y0090), Shanghai Rising-Star Program (18QA1404100), Program for Professor of Special Appointment (Eastern Scholar) at Shanghai Institutions of Higher Learning, Shanghai Youth Talent Program, Program of Shanghai Academic/Technology Research Leader (18XD1403800), Shanghai Municipal Key Clinical Specialty (shslczdzk03701), The Three-Year Plan of Shanghai Municipality for Further Accelerating The Development of Traditional Chinese Medicine (ZY(2018-2020)-CCCX-1016), Shanghai Science and Technology Development Fund (20JC1411300), Shanghai Chenguang Program (18CG47), Gaofeng Clinical Medicine Grant of Shanghai Municipal Education Commission, Shanghai Frontier Research Base of Disease and Syndrome Biology of Inflammatory cancer transformation (2021KJ03-12), Health Commission of Pudong New Area Health and Family Planning Scientific Research Project (PW2019E-1), and Xinling Scholar Program of Shanghai University of Traditional Chinese Medicine. In addition, we appreciate that Dr. Chenying Liu (Xinhua Hospital affiliated to Shanghai Jiao Tong University, Shanghai 200092, China) provided critical comments for our manuscript.

AUTHOR CONTRIBUTIONS

W.-L.C and L.J. conceptualized the study. D.L., X.J., M.W., W.Z., J.W., J.Y., F.W., L.C., Q.L., X.K., Y.Q., and Z.X. performed *in vitro* and *in vivo* studies. D.L. and X.J. conducted the metabolomics assays. G.Y., L.L., and X.Y. harvested human cancer samples. W.-L.C and D.L. wrote the original draft. W.-L.C. and L.J. reviewed and edited the manuscript.

DECLARATION OF INTERESTS

The authors declare no competing interests.

REFERENCES

1. Siegel, R.L., Miller, K.D., Fuchs, H.E., and Jemal, A. (2021). Cancer Statistics, 2021. CA Cancer J. Clin. *71*, 7–33.
2. Wang, J.J., Lei, K.F., and Han, F. (2018). Tumor microenvironment: recent advances in various cancer treatments. Eur. Rev. Med. Pharmacol. Sci. *22*, 3855–3864.
3. Attar, R., Tabassum, S., Fayyaz, S., Ahmad, M.S., Nogueira, D.R., Yaylim, I., Timirci-Kahraman, O., Kucukhuseyin, O., Cacina, C., Farooqi, A.A., and Ismail, M. (2015). Natural products are the future of anticancer therapy: Preclinical and clinical advancements of Viscum album phytometabolites. Cell. Mol. Biol. *61*, 62–68.
4. Zupkó, I., Jaeger, W., Topcu, Z., and Wu, C.C. (2015). Anticancer Properties of Natural Products. BioMed Res. Int. *2015*, 242070.
5. Patlolla, J.M., and Rao, C.V. (2012). Triterpenoids for cancer prevention and treatment: current status and future prospects. Curr. Pharm. Biotechnol. *13*, 147–155.
6. Ying, H., Kimmelman, A.C., Lyssiotis, C.A., Hua, S., Chu, G.C., Fletcher-Sananikone, E., Locasale, J.W., Son, J., Zhang, H., Coloff, J.L., et al. (2012). Oncogenic Kras maintains pancreatic tumors through regulation of anabolic glucose metabolism. Cell *149*, 656–670.
7. Gu, X., Xu, Z.Y., Zhu, L.Y., Wang, L.F., Li, K., and Pei, Q. (2013). Dual control of Shuanghuang Shengbai granule on upstream and downstream signal modulators of CyclinD-CDK4/6 signaling pathway of cell cycle in Lewis-bearing mice with cyclophosphamide-induced myelosuppression. OncoTargets Ther. *6*, 199–209.
8. Liao, S.G., Wang, Z., Wu, Y.Y., Zhang, L.J., Li, J., Wang, A.M., Li, Y.J., Lan, Y.Y., and Wang, Y.L. (2014). Report: structures and hepatocytotoxicity of co-occurring substances in oleanolic acid tablets. Pak. J. Pharm. Sci. *27*, 601–605.
9. Liu, J., Zheng, L., Wu, N., Ma, L., Zhong, J., Liu, G., and Lin, X. (2014). Oleanolic acid induces metabolic adaptation in cancer cells by activating the AMP-activated protein kinase pathway. J. Agric. Food Chem. *62*, 5528–5537.
10. Liu, J., Ma, L., Chen, X., Wang, J., Yu, T., Gong, Y., Ma, A., Zheng, L., and Liang, H. (2016). ERK inhibition sensitizes cancer cells to oleanolic acid-induced apoptosis through ERK/Nrf2/ROS pathway. Tumour Biol. *37*, 8181–8187.
11. Vander Heiden, M.G., and DeBerardinis, R.J. (2017). Understanding the Intersections between Metabolism and Cancer Biology. Cell *168*, 657–669.
12. Kroemer, G., and Pouyssegur, J. (2008). Tumor cell metabolism: cancer's Achilles' heel. Cancer Cell *13*, 472–482.
13. Intlekofer, A.M., and Finley, L.W.S. (2019). Metabolic signatures of cancer cells and stem cells. Nat. Metab. *1*, 177–188.
14. Chen, W.L., Wang, Y.Y., Zhao, A., Xia, L., Xie, G., Su, M., Zhao, L., Liu, J., Qu, C., Wei, R., et al. (2016). Enhanced Fructose Utilization Mediated by SLC2A5 Is a Unique Metabolic Feature of Acute Myeloid Leukemia with Therapeutic Potential. Cancer Cell *30*, 779–791.
15. Faubert, B., Li, K.Y., Cai, L., Hensley, C.T., Kim, J., Zacharias, L.G., Yang, C., Do, Q.N., Doucette, S., Burguete, D., et al. (2017). Lactate Metabolism in Human Lung Tumors. Cell *171*, 358–371.e9.
16. Mayers, J.R., Torrence, M.E., Danai, L.V., Papagiannakopoulos, T., Davidson, S.M., Bauer, M.R., Lau, A.N., Ji, B.W., Dixit, P.D., Hosios, A.M., et al. (2016). Tissue of origin dictates branched-chain amino acid metabolism in mutant Kras-driven cancers. Science *353*, 1161–1165.
17. Lane, A.N., and Fan, T.W. (2015). Regulation of mammalian nucleotide metabolism and biosynthesis. Nucleic Acids Res. *43*, 2466–2485.
18. Yin, J., Ren, W., Huang, X., Deng, J., Li, T., and Yin, Y. (2018). Potential Mechanisms Connecting Purine Metabolism and Cancer Therapy. Front. Immunol. *9*, 1697.

19. Sreekumar, A., Poisson, L.M., Rajendiran, T.M., Khan, A.P., Cao, Q., Yu, J., Laxman, B., Mehra, R., Lonigro, R.J., Li, Y., et al. (2009). Metabolomic profiles delineate potential role for sarcosine in prostate cancer progression. Nature *457*, 910–914.

20. Chen, W.L., Wang, J.H., Zhao, A.H., Xu, X., Wang, Y.H., Chen, T.L., Li, J.M., Mi, J.Q., Zhu, Y.M., Liu, Y.F., et al. (2014). A distinct glucose metabolism signature of acute myeloid leukemia with prognostic value. Blood *124*, 1645–1654.

21. Rawendra, R.D., Lin, P.Y., Chang, C.D., Hsu, J.L., Huang, T.C., and Shih, W.L. (2015). Potentiation of Acute Promyelocytic Leukemia Cell Differentiation and Prevention of Leukemia Development in Mice by Oleanolic Acid. Anticancer Res. *35*, 6583–6590.

22. Ward, P.S., and Thompson, C.B. (2012). Metabolic reprogramming: a cancer hallmark even warburg did not anticipate. Cancer Cell *21*, 297–308.

23. Tennant, D.A., Durán, R.V., and Gottlieb, E. (2010). Targeting metabolic transformation for cancer therapy. Nat. Rev. Cancer *10*, 267–277.

24. Hakimi, A.A., Reznik, E., Lee, C.H., Creighton, C.J., Brannon, A.R., Luna, A., Aksoy, B.A., Liu, E.M., Shen, R., Lee, W., et al. (2016). An Integrated Metabolic Atlas of Clear Cell Renal Cell Carcinoma. Cancer Cell *29*, 104–116.

25. Hajieva, P. (2017). The Effect of Polyphenols on Protein Degradation Pathways: Implications for Neuroprotection. Molecules *22*, 159–171.

26. Schneider-Poetsch, T., Ju, J., Eyler, D.E., Dang, Y., Bhat, S., Merrick, W.C., Green, R., Shen, B., and Liu, J.O. (2010). Inhibition of eukaryotic translation elongation by cycloheximide and lactimidomycin. Nat. Chem. Biol. *6*, 209–217.

27. Hofmann, K., and Falquet, L. (2001). A ubiquitin-interacting motif conserved in components of the proteasomal and lysosomal protein degradation systems. Trends Biochem. Sci. *26*, 347–350.

28. Mauthe, M., Orhon, I., Rocchi, C., Zhou, X., Luhr, M., Hijlkema, K.J., Coppes, R.P., Engedal, N., Mari, M., and Reggiori, F. (2018). Chloroquine inhibits autophagic flux by decreasing autophagosome-lysosome fusion. Autophagy *14*, 1435–1455.

29. Mauvezin, C., and Neufeld, T.P. (2015). Bafilomycin A1 disrupts autophagic flux by inhibiting both V-ATPase-dependent acidification and Ca-P60A/SERCA-dependent autophagosome-lysosome fusion. Autophagy *11*, 1437–1438.

30. Davidson, S.M., and Vander Heiden, M.G. (2017). Critical Functions of the Lysosome in Cancer Biology. Annu. Rev. Pharmacol. Toxicol. *57*, 481–507.

31. Hardie, D.G. (2011). AMPK and autophagy get connected. EMBO J. *30*, 634–635.

32. Mizushima, N., and Yoshimori, T. (2007). How to interpret LC3 immunoblotting. Autophagy *3*, 542–545.

33. Herzig, S., and Shaw, R.J. (2018). AMPK: guardian of metabolism and mitochondrial homeostasis. Nat. Rev. Mol. Cell Biol. *19*, 121–135.

34. Martin, K.R., Celano, S.L., Solitro, A.R., Gunaydin, H., Scott, M., O'Hagan, R.C., Shumway, S.D., Fuller, P., and MacKeigan, J.P. (2018). A Potent and Selective ULK1 Inhibitor Suppresses Autophagy and Sensitizes Cancer Cells to Nutrient Stress. iScience *8*, 74–84.

35. Petherick, K.J., Conway, O.J., Mpamhanga, C., Osborne, S.A., Kamal, A., Saxty, B., and Ganley, I.G. (2015). Pharmacological inhibition of ULK1 kinase blocks mammalian target of rapamycin (mTOR)-dependent autophagy. J. Biol. Chem. *290*, 11376–11383.

36. Park, J.M., Jung, C.H., Seo, M., Otto, N.M., Grunwald, D., Kim, K.H., Moriarity, B., Kim, Y.M., Starker, C., Nho, R.S., et al. (2016). The ULK1 complex mediates MTORC1 signaling to the autophagy initiation machinery via binding and phosphorylating ATG14. Autophagy *12*, 547–564.

37. Levy, J.M.M., Towers, C.G., and Thorburn, A. (2017). Targeting autophagy in cancer. Nat. Rev. Cancer *17*, 528–542.

38. Madrigal-Matute, J., and Cuervo, A.M. (2016). Regulation of Liver Metabolism by Autophagy. Gastroenterology *150*, 328–339.

39. Oshima, M., Seki, T., Kurauchi, Y., Hisatsune, A., and Katsuki, H. (2019). Reciprocal Regulation of Chaperone-Mediated Autophagy/Microautophagy and Exosome Release. Biol. Pharm. Bull. *42*, 1394–1401.

40. Eichner, L.J., Brun, S.N., Herzig, S., Young, N.P., Curtis, S.D., Shackelford, D.B., Shokhirev, M.N., Leblanc, M., Vera, L.I., Hutchins, A., et al. (2019). Genetic Analysis Reveals AMPK Is Required to Support Tumor Growth in Murine Kras-Dependent Lung Cancer Models. Cell Metab. *29*, 285–302.e7.

41. Wei, J., Liu, M., Liu, H., Wang, H., Wang, F., Zhang, Y., Han, L., and Lin, X. (2013). Oleanolic acid arrests cell cycle and induces apoptosis via ROS-mediated mitochondrial depolarization and lysosomal membrane permeabilization in human pancreatic cancer cells. J. Appl. Toxicol. *33*, 756–765.

42. Rabinovitch, R.C., Samborska, B., Faubert, B., Ma, E.H., Gravel, S.P., Andrzejewski, S., Raissi, T.C., Pause, A., St-Pierre, J., and Jones, R.G. (2017). AMPK Maintains Cellular Metabolic Homeostasis through Regulation of Mitochondrial Reactive Oxygen Species. Cell Rep. *21*, 1–9.

43. Hardie, D.G. (2014). AMPK–sensing energy while talking to other signaling pathways. Cell Metab. *20*, 939–952.

44. Liou, G.Y., and Storz, P. (2010). Reactive oxygen species in cancer. Free Radic. Res. *44*, 479–496.

45. Somwar, R., Erdjument-Bromage, H., Larsson, E., Shum, D., Lockwood, W.W., Yang, G., Sander, C., Ouerfelli, O., Tempst, P.J., Djaballah, H., and Varmus, H.E. (2011). Superoxide dismutase 1 (SOD1) is a target for a small molecule identified in a screen for inhibitors of the growth of lung adenocarcinoma cell lines. Proc. Natl. Acad. Sci. USA *108*, 16375–16380.

46. Tang, Z., Kang, B., Li, C., Chen, T., and Zhang, Z. (2019). GEPIA2: an enhanced web server for large-scale expression profiling and interactive analysis. Nucleic Acids Res. *47* (W1), W556–W560.

47. Guo, W., Tan, H.Y., Li, S., Wang, N., and Feng, Y. (2020). Glutamic-Pyruvic Transaminase 1 Facilitates Alternative Fuels for Hepatocellular Carcinoma Growth-A Small Molecule Inhibitor, Berberine. Cancers (Basel) *12*, 1854–1871.

48. Tong, X., Zhao, F., and Thompson, C.B. (2009). The molecular determinants of de novo nucleotide biosynthesis in cancer cells. Curr. Opin. Genet. Dev. *19*, 32–37.

49. Villa, E., Ali, E.S., Sahu, U., and Ben-Sahra, I. (2019). Cancer Cells Tune the Signaling Pathways to Empower de Novo Synthesis of Nucleotides. Cancers (Basel) *11*, 688.

50. Townsend, M.H., Robison, R.A., and O'Neill, K.L. (2018). A review of HPRT and its emerging role in cancer. Med. Oncol. *35*, 89.

51. Jiang, T., Xu, X., Qiao, M., Li, X., Zhao, C., Zhou, F., Gao, G., Wu, F., Chen, X., Su, C., et al. (2018). Comprehensive evaluation of NT5E/CD73 expression and its prognostic significance in distinct types of cancers. BMC Cancer *18*, 267.

52. Papa, L., Manfredi, G., and Germain, D. (2014). SOD1, an unexpected novel target for cancer therapy. Genes Cancer *5*, 15–21.

53. Gonzalez, P.S., O'Prey, J., Cardaci, S., Barthet, V.J.A., Sakamaki, J.I., Beaumatin, F., Roseweir, A., Gay, D.M., Mackay, G., Malviya, G., et al. (2018). Mannose impairs tumour growth and enhances chemotherapy. Nature *563*, 719–723.

54. Qiu, Y., Cai, G., Zhou, B., Li, D., Zhao, A., Xie, G., Li, H., Cai, S., Xie, D., Huang, C., et al. (2014). A distinct metabolic signature of human colorectal cancer with prognostic potential. Clin. Cancer Res. *20*, 2136–2146.

55. Svensson, R.U., Parker, S.J., Eichner, L.J., Kolar, M.J., Wallace, M., Brun, S.N., Lombardo, P.S., Van Nostrand, J.L., Hutchins, A., Vera, L., et al. (2016). Inhibition of acetyl-CoA carboxylase suppresses fatty acid synthesis and tumor growth of non-small-cell lung cancer in preclinical models. Nat. Med. *22*, 1108–1119.

56. Joung, J., Konermann, S., Gootenberg, J.S., Abudayyeh, O.O., Platt, R.J., Brigham, M.D., Sanjana, N.E., and Zhang, F. (2017). Genome-scale CRISPR-Cas9 knockout and transcriptional activation screening. Nat. Protoc. *12*, 828–863.

57. Liu, Z.W., Zhu, H.T., Chen, K.L., Dong, X., Wei, J., Qiu, C., and Xue, J.H. (2013). Protein kinase RNA-like endoplasmic reticulum kinase (PERK) signaling pathway plays a major role in reactive oxygen species (ROS)-mediated endoplasmic reticulum stress-induced apoptosis in diabetic cardiomyopathy. Cardiovasc. Diabetol. *12*, 158.

58. Chen, W.L., Jin, X., Wang, M., Liu, D., Luo, Q., Tian, H., Cai, L., Meng, L., Bi, R., Wang, L., et al. (2020). GLUT5-mediated fructose utilization drives lung cancer growth by stimulating fatty acid synthesis and AMPK/mTORC1 signaling. JCI Insight *5*, e131596.

59. Yu, G., Yu, W., Jin, G., Xu, D., Chen, Y., Xia, T., Yu, A., Fang, W., Zhang, X., Li, Z., and Xie, K. (2015). PKM2 regulates neural invasion of and predicts poor prognosis for human hilar cholangiocarcinoma. Mol. Cancer *14*, 193.

60. Rohart, F., Gautier, B., Singh, A., and Lê Cao, K.A. (2017). mixOmics: An R package for 'omics feature selection and multiple data integration. PLoS Comput. Biol. *13*, e1005752.

OMTO, Volume *23*

Supplemental information

Oleanolic acid blocks the purine salvage pathway for cancer therapy by inactivating SOD1 and stimulating lysosomal proteolysis

Dan Liu, Xing Jin, Guanzhen Yu, Mingsong Wang, Lei Liu, Wenjuan Zhang, Jia Wu, Fengying Wang, Jing Yang, Qin Luo, Lili Cai, Xi Yang, Xisong Ke, Yi Qu, Zhenye Xu, Lijun Jia, and Wen-Lian Chen

Supplemental information

Supplemental Materials and Methods

Apoptosis assay

A549 and MDA-MB-231 cells were seeded into 6-well plates at a density of 1.5×10^5/well, and then treated by 200 μM OA for 0, 24, or 48 hours. Spent medium of each well was collected individually to harvest cells by centrifuging at 1500 rpm for 5 minutes, while adherent cells of each well were digested with 0.5% trypsin and harvested. For each well, cells from spent medium and adherent cells were combined together and then washed by PBS for 2 times. Finally, cell apoptosis assay was implemented on a flow cytometer (BD FACSVerse™, New Jersey, USA) using an AnnexinV-FITC and PI Apoptosis Kit (Yuheng Biotechnology, Suzhou, China) according to the manufacturer's instructions.

Cell cycle analysis

A549 and MDA-MB-231 cells were seeded into 6-well plates at a density of 1.5×10^5/well and treated by 200 μM OA for 0, 24, or 48 hours. After removal of the spent medium, OA-treated cells and control cells were harvested and fixed with 70% ethanol at -20 °C overnight. Subsequently, the fixed cells were stained with propidium iodide (PI) (Sigma, St. Louis, MO, USA) at a concentration of 36 mg/mL at 37 °C for 15 minutes. Next, these cells were used for fluorescence activated cell sorting (FACS) analysis by a flow cytometer (BD FACSVerse™, New Jersey, USA). Data were analyzed with FlowJo 7.6 software.

Co-immunoprecipitation and ubiquitination assays of HGPRT

For the co-IP assay of HGPRT, A549 cells treated by vehicle or 200 μM OA for 8 hours were harvested and lysed in NP40 buffer (Beyotime, Shanghai, China) for 30 minutes on ice, and then centrifuged at 13,000 ×g for 15 minutes at 4 ℃. The lysates were incubated with anti-rabbit IgG (Cell Signaling Technology, Boston, Massachusetts, USA) or HGPRT antibody (ab109021, Abcam, Cambridge, UK) at 4 ℃ with rocking overnight. Subsequently, the lysates were incubated with protein A/G PLUS-Agarose beads (Santa Cruz, Dallas, Texas, USA) at 4 ℃ for 2 hours. The proteins binding to the beads were washed for three times by using NP40 buffer and then boiled for 10 minutes at 95 ℃, followed by SDS-PAGE, and finally analyzed by immunoblotting with primary antibodies against human hypoxanthine-guanine phosphoribosyltransferase (ab109021, Abcam, Cambridge, UK) and ubiquitin (Santa Cruz, Dallas, Texas, USA).

Table S1. Clinical characteristics of lung cancer patient cohort 1.

	Lung adenocarcinoma (n=5)
Age, years	
Median	64
Range	58-68
Gender, no. (%)	
Male	2（40）
Female	3（60）
TNM stage, no. (%)	
1	2（40）
2	2（40）
3	1（20）

Table S2. Clinical characteristics of lung cancer patient cohort 2.

	Lung adenocarcinoma (n=17)	Lung squamous cell carcinoma (n=17)
Age, years		
Median	60	60
Range	41-75	43-78
Gender, no. (%)		
Male	11（64.7）	14（82.3）
Female	6（35.3）	3（17.6）
TNM stage, no. (%)		
1	5（29.4）	
2	6（35.3）	7（41.2）
3	4（23.5）	5（29.4）
4	1（5.9）	1（5.9）
Unclassified	1（5.9）	4（23.5）

Table S3. Clinical characteristics of breast cancer patient cohort.

	Luminal-like (n=10)	Triple Negative (n=1)	HER2 positive (n=1)	Unclassified (n=8)
Age, years				
Median	52	49	59	47
Range	36-72	49	59	37-69
Gender, no. (%)				
Female	10（100）	1（100）	1（100）	8（100）
TNM stage, no. (%)				
1	3（30）	0（0）	0（0）	0（0）
2	5（50）	1（100）	1（100）	3（37.5）
3	2（20）	0（0）	0（0）	4（50.0）
4	0（0）	0（0）	0（0）	0（0）
Unclassified	0（0）	0（0）	0（0）	1（12.5）

Table S4. Clinical characteristics of lung cancer patient cohort 3.

	Lung adenocarcinoma (n=21)	Lung squamous cell carcinoma (n=13)
Age, years		
Median	60	63
Range	42-79	58-77
Gender, no. (%)		
Male	10（47.6）	11（84.6）
Female	11（52.4）	2（15.4）
TNM stage, no. (%)		
1	1（4.8）	0
2	13（61.9）	10（76.9）
3	6（28.5）	3（23.1）
4	1（4.8）	0

Table S5. Sequences of gene-specific q-PCR primers in this study.

Gene	NCBI Lous ID	Forward primer sequence (5'-3')	Reverse primer sequence (5'-3')
HPRT1	NM_3251	CCTGGCGTCGTGATTAGTGAT	AGACGTTCAGTCCTGTCCATAA
NT5E	NM_4907	AAGGACTGATCGAGCCACTC	GGAAGTGTATCCAACGATTCCCA
ACTIN	NM_001101.3	CACTCTTCCAGCCTTCCTTC	GTACAGGTCTTTGCGGATGT

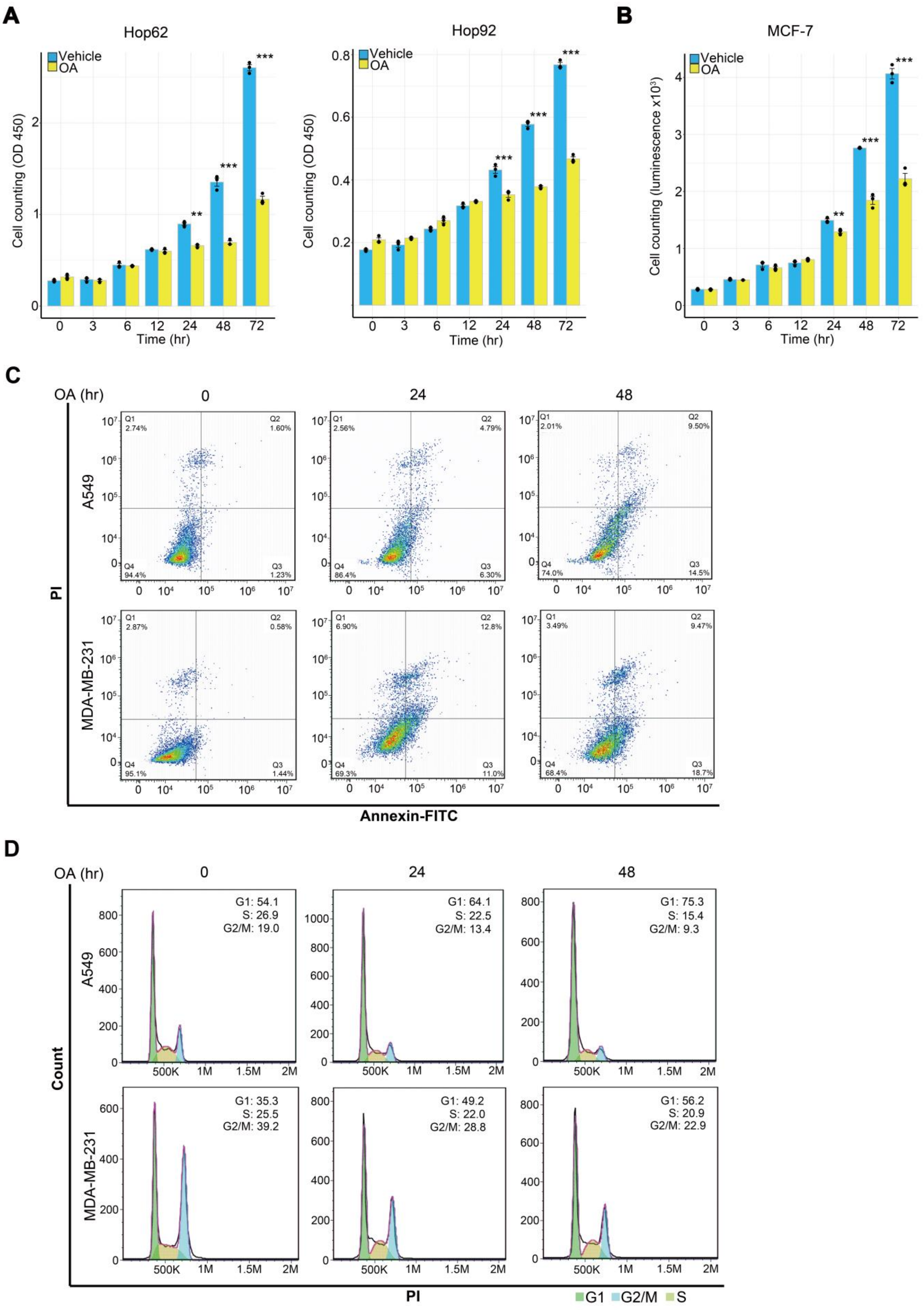
A
Hop62
Vehicle
OA
Cell counting (OD 450)
Time (hr)
Hop92
B
MCF-7
Cell counting (luminescence x10³)
C
OA (hr)
A549
MDA-MB-231
PI
Annexin-FITC
D
G1: 54.1
S: 26.9
G2/M: 19.0
G1: 64.1
S: 22.5
G2/M: 13.4
G1: 75.3
S: 15.4
G2/M: 9.3
G1: 35.3
S: 25.5
G2/M: 39.2
G1: 49.2
S: 22.0
G2/M: 28.8
G1: 56.2
S: 20.9
G2/M: 22.9
Count
G1 G2/M S

Figure S1 Oleanolic acid impedes cancer cell growth and induces cell apoptosis and cell cycle arrest *in vitro*.

(A-B) The time-course influence of 200 μM oleanolic acid (OA) on the cell proliferation of two lung cancer cell lines Hop92 and Hop62, and one breast cancer cell line MCF-7. **(C)** The time course impact of 200 μM OA on cell apoptosis of A549 and MDA-MB-231 cells. **(D)** The time course effect of 200 μM OA on cell cycle of A549 and MDA-MB-231 cells.

Error bars represent mean ± SEM. $^{*}p < 0.05$, $^{**}p < 0.01$, $^{***}p < 0.001$, Student's t test.

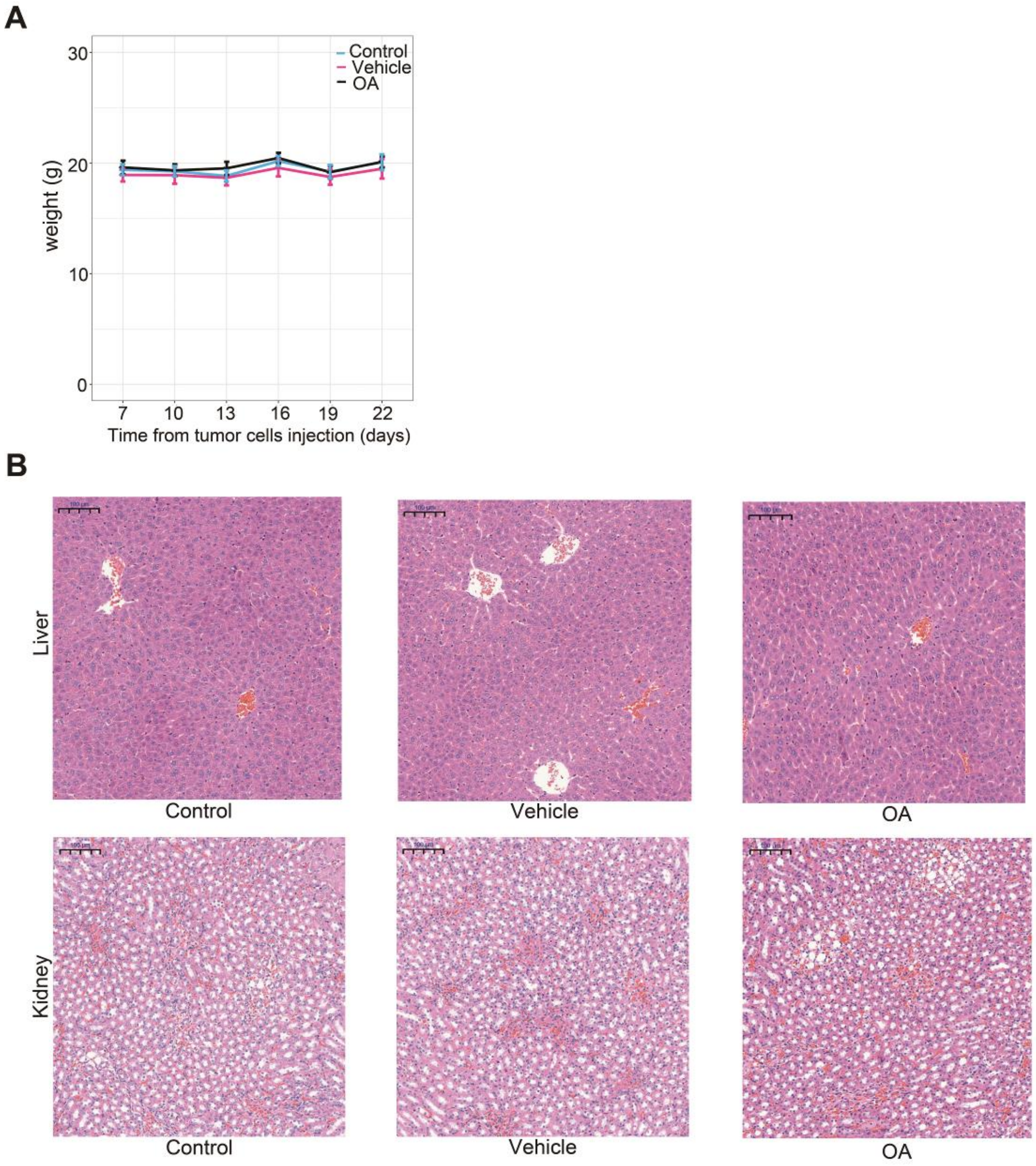

Figure S2. The influence of oleanolic acid treatment on mouse body weight and histological structure of liver and kidney.

(A) Body weight of healthy control mice, A549 tumor-bearing mice treated with vehicle, and A549 tumor-bearing mice treated with oleanolic acid (OA, 120 mg/kg/day). Error bars represent mean ± SEM. **(B)** Histological structure of liver and kidney tissues from healthy control mice, A549 tumor-bearing mice treated with vehicle, and A549 tumor-bearing mice treated with OA (120 mg/kg/day). Scale bars, 100 µm.

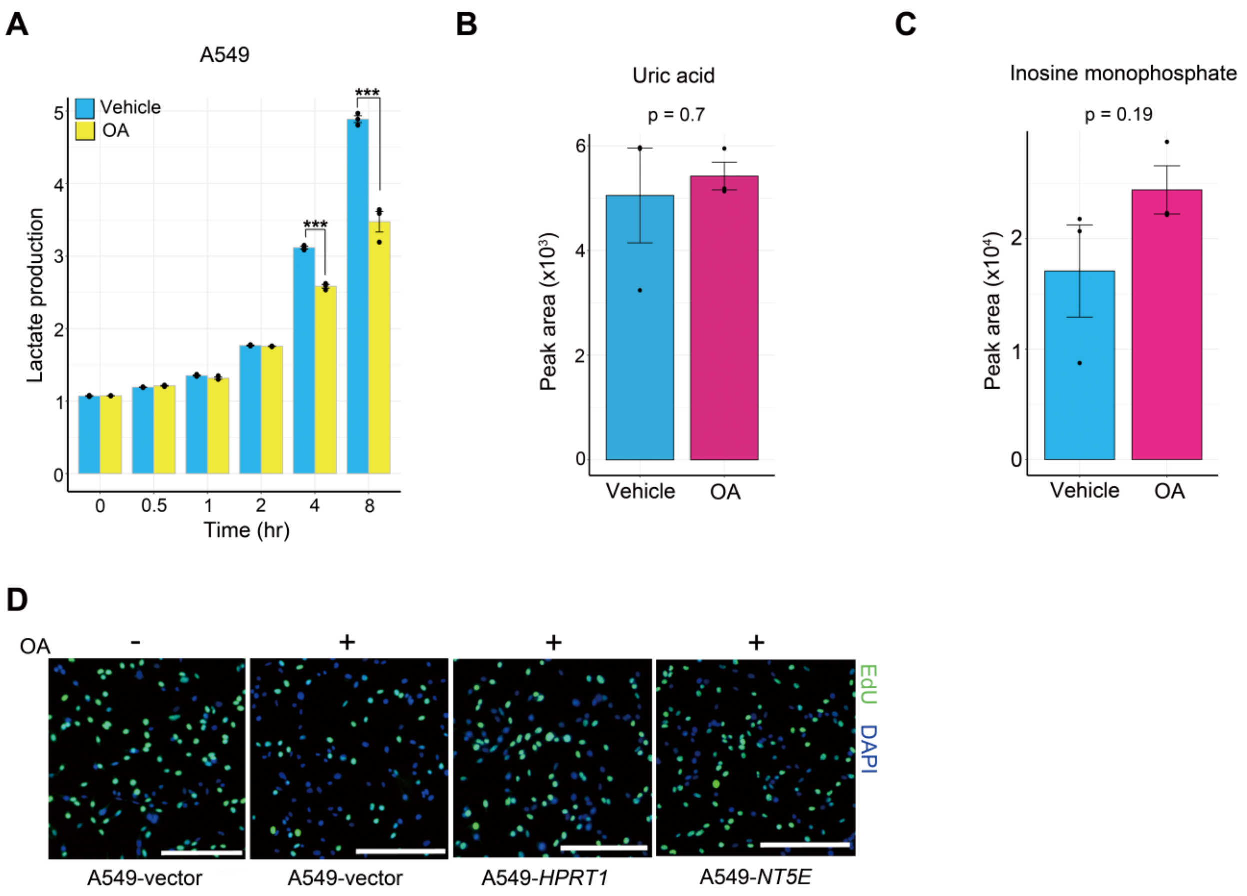

Figure S3. The impact of oleanolic acid treatment on cancer cell metabolism and the rescue effect of enforced expression of *HPRT1* or *NT5E* on DNA synthesis suppressed by oleanolic acid in A549 cells.

(A) The time course assay of lactate production of A549 cells treated by 200 μM oleanolic acid (OA). **(B)** Uric acid generation between A549 cells treated by vehicle (1/1000 DMSO) and A549 cells treated by 200 μM OA for 8 hours. **(C)** Inosine monophosphate (IMP) production between A549 cells treated by vehicle (1/1000 DMSO) and A549 cells treated by 200 μM OA for 8 hours. **(D)** DNA synthesis was remarkably refrained in A549-vector cells treated by 200 μM OA for 8 hours as compared to A549-vector cells treated by vehicle. Of note, impaired DNA synthesis elicited by OA was overtly rescued by forced expression of *HPRT1* or *NT5E*. DNA synthesis was measured by EdU incorporation (green), while cell nuclei were stained by DAPI (blue). Scale bars, 100 μm.

Error bars represent mean ± SEM. $^{*}p < 0.05$, $^{**}p < 0.01$, $^{***}p < 0.001$, Student's t test.

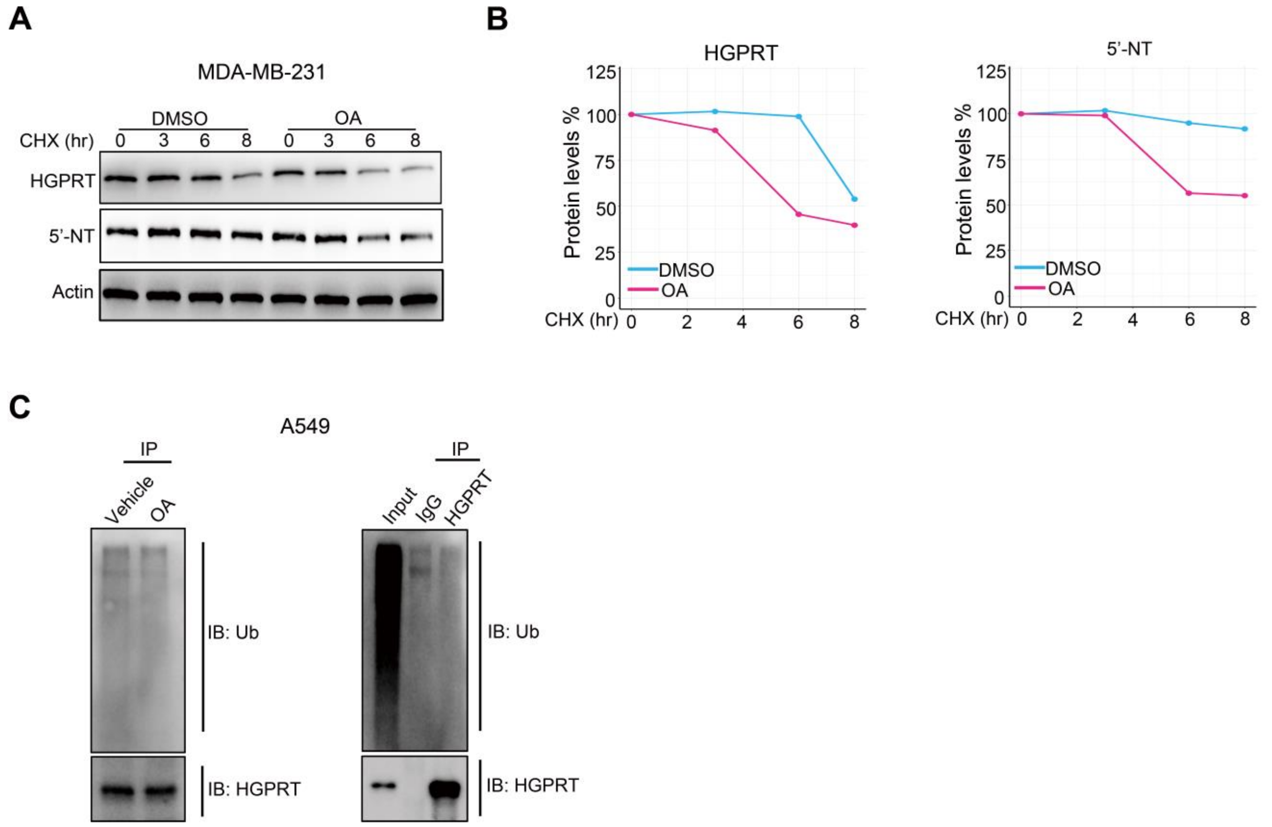

Figure S4. Oleanolic acid expedites the degradation of PSP enzymes via lysosomal pathway but not ubiquitination pathway.

(A-B) The time-course influence of 200 μM oleanolic acid (OA) treatment on HGRPT and 5'-NT degradation when using cycloheximide (CHX, 50 μg/mL) to block protein synthesis in MDA-MB-231 cells. The curves on the right side (B) displaying the quantification results of the protein levels across different time points. **(C)** The effect of 200 μM OA treatment for 8 hours on the ubiquitination status of HGPRT in A549 cells. The affinity and specificity of the anti-HGPRT antibody to cellular HGPRT protein was validated as showed by the immunoblots on the right side.

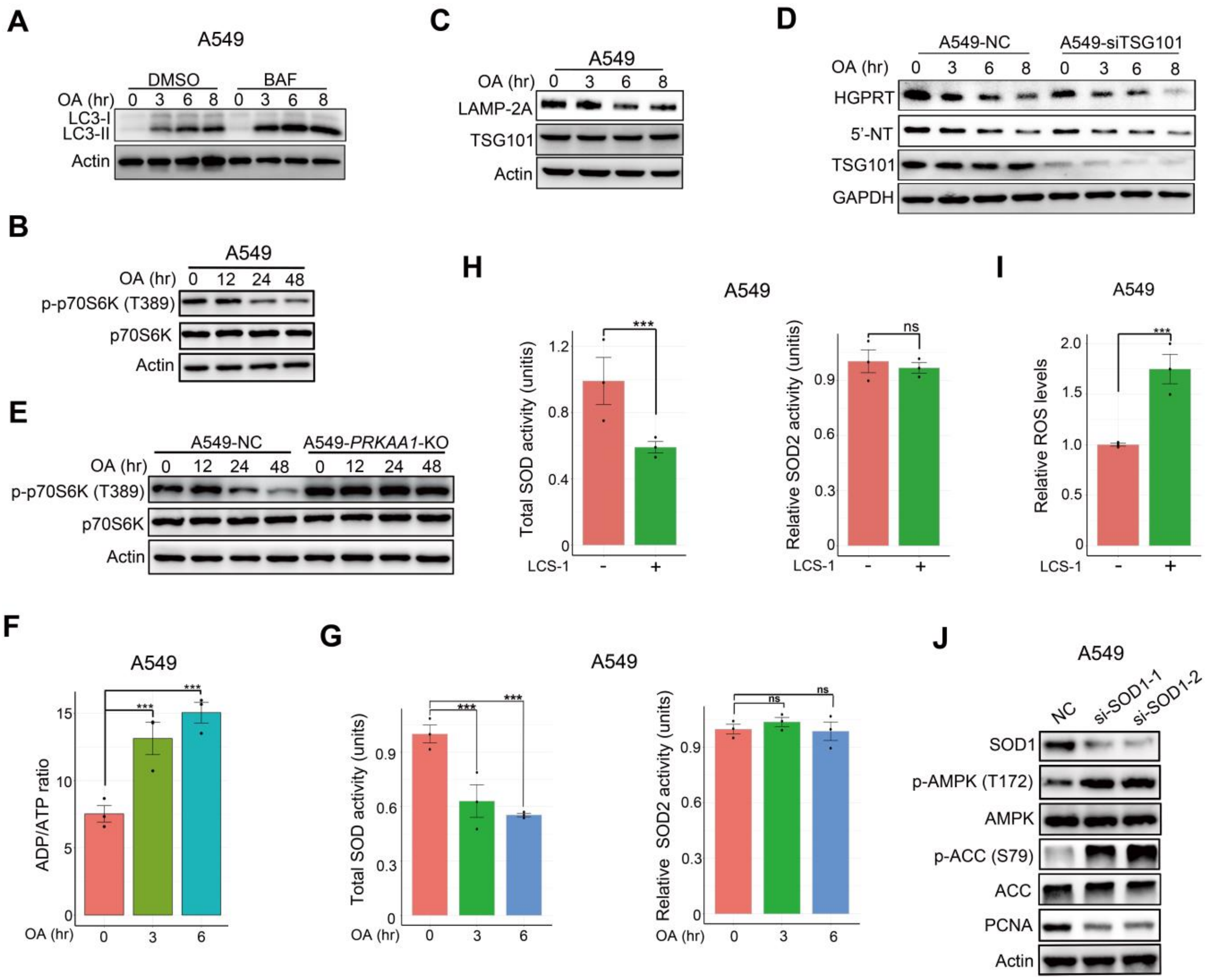

Figure S5. Stimulation of macroautophagy but not microautophagy and chaperone-mediated autophagy by oleanolic acid treatment, and the underlying mechanism of oleanolic acid-activated macroautophagy.

(A) Western blot showing the time course of a lysosome inhibitor bafilomycin A1 (BAF, 0.08 μM) treatment effect on LC3-I/II levels in A549 cells under the condition of 200 μM OA treatment. **(B)** Western blot showing the time course of the impact of 200 μM OA treatment on the expression of phospho-p70S6K (T389) and p70S6K in A549 cells. **(C)** Western blot showing the time course of the effect of 200 μM OA treatment on the expression of TSG101 and LAMP-2A in A549 cells. TSG101 is a microautophagy-related protein, while LAMP-2A is a chaperone-mediated

autophagy-related protein. **(D)** Western blot showing the time course of the effect of 200 μM OA treatment on the expression of HGPRT and 5’-NT between control A549 cells and A549 cells with TSG101 silencing. TSG101 knockdown was used to inhibit microautophagy. **(E)** Western blot showing the time course of the effect of 200 μM OA treatment on the expression of phosho-p70S6K (T389) and p70S6K in A549 cells with and without *PRKAA1*-KO. **(F)** The time course of the effect of 200 μM OA treatment on ADP/ATP ratio in A549 cells. **(G)** The time course of the influence of 200 μM OA treatment on the activities of total superoxide dismutases (SODs) and SOD2 in A549 cells. **(H)** The impact of a SOD1 inhibitor LCS-1 (5 μM**)** treatment for 6 hours on the activity of total SODs and SOD2 in A549 cells. **(I)** Measurement of ROS generation in A549 cells with or without LCS-1 (5 μM) treatment for 6 hours. **(J)** Western blot showing the impact of SOD1 silencing on the expression of SOD1, phospho-AMPK (T172), AMPK, phospho-ACC (S79), ACC and PCNA in A549 cells. The cells were treated by siRNAs for 72 hours. NC, nontarget control siRNA sequence. Error bars represent mean ± SEM. *$p < 0.05$, **$p < 0.01$, ***$p < 0.001$, Student’s t test. Ns, no significance.
